# Supplementary material for: Rapid inverse design of metamaterials based on prescribed mechanical behavior through machine learning
Source: Nat Commun. 2023 Sep 18;14:5765. doi: 10.1038/s41467-023-40854-1 (PMC10505607; doi:10.1038/s41467-023-40854-1)
Supplement: Supplementary file 1 — Supplementary Information [file 41467_2023_40854_MOESM1_ESM.pdf]

Supplementary Information for

Rapid Inverse Design of Metamaterials based on Prescribed Mechanical Behavior through Machine Learning

Authors: *Chan Soo Ha[1,†], Desheng Yao[2,3†], Zhenpeng Xu[2,3], Chenang Liu[4], Han Liu[5], Daniel Elkins[1,6], Matt Kile[1], Vikram Deshpande[7*], Zhenyu Kong[6,*], Mathieu Bauchy[3*], and Xiaoyu (Rayne) Zheng[1,2,3*]*

Author Affiliations:

[1]Department of Mechanical Engineering, Virginia Tech, Blacksburg, VA, USA

[2]Department of Material Science and Engineering, University of California, Berkeley, CA, USA

[3]Department of Civil and Environmental Engineering, University of California, Los Angeles, CA, USA

[4]Industrial Engineering and Management, Oklahoma State University, Stillwater, OK, USA

[5]Department of Computer Science and Technology, Sichuan University, Chengdu, China

[6]Grado Department of Industrial and Systems Engineering, Virginia Tech, Blacksburg, VA, USA

[7]Department of Engineering, University of Cambridge, Cambridge, UK

[†]These authors contributed equally to this work.

[*]**Corresponding authors:** X. Zheng, Email: rayne@seas.ucla.edu; Z. Kong, Email: zkong@vt.edu; M. Bauchy, Email: bauchy@ucla.edu; V. Deshpande, Email: vsd20@cam.ac.uk

This Supplementary Information includes:

Supplementary Note 1. Machine learning model structure
Supplementary Note 2. Derivation of stress-strain curve design space
Supplementary Note 3. Plottable stress-strain curve paths
Supplementary Note 4. Stress-strain curve parameterization
Supplementary Note 5. Choice of architectural unit cells
Supplementary Note 6. Generation of training instances
Supplementary Note 7. Training of the machine learning
Supplementary Note 8. Study of process variability
Supplementary Note 9. Inverse design of architected shoe midsole
Supplementary Note 10. Inverse design of compound lattices
Supplementary Note 11. Details of FE simulations
Supplementary Note 12. Applicability of our ML pipeline to other mechanical loadings

Additional Supplementary Files:

Supplementary Movie 1. Architectural evolution of the cubic symmetric, strut-based unit cells (available online)

Supplementary Movie 2. In situ movie of the inversely designed sample shown in Fig. 5d in response to uniaxial cyclic compression (available online)

Supplementary Movie 3. In situ movie of the inversely designed sample shown in Fig. 5e in response to uniaxial cyclic compression (available online)

Supplementary Movie 4. In situ movie of the inversely designed sample shown in Fig. 5f in response to uniaxial cyclic compression (available online)

Supplementary Note 1. Machine learning model structure

Supplementary Note 1.1. Generative machine learning pipeline

By inputting the target stress-strain curve feature $\{\mathbf{X}^{T}\}$, we aimed to build an inverse (backward) model that predicts the design parameters $\{\mathbf{Y}\}$ of a lattice architecture, which is capable replicating the targeted curve after fabricating and compression testing. In general, the inverse prediction from $\{\mathbf{X}^{T}\}$ to $\{\mathbf{Y}\}$ can theoretically be affected by the "one-to-many" issue, that is, although a given lattice architecture presents a unique stress-strain curve, several lattice architectures can potentially provide similar or same stress-strain curve. Therefore, the conventional inverse model's loss function $f_L=(\mathbf{Y}_{pred}-\mathbf{Y}_{true})^2$ (where $\mathbf{Y}$ is the design parameters of the designed lattice architecture) is ill-defined since several ground-truth values of $\mathbf{Y}_{true}$ are correct—that is, several lattice architectures $\{\mathbf{Y}\}$ can exhibit the same feature $\{\mathbf{X}\}$ of the stress-strain curve. The ill-defined nature of the cost function is an issue as it serves as the basis (i.e., cost function) for the training of the model.

Here, we implemented a generative machine learning model to address the "one-to-many" issue. Supplementary Fig. 1a illustrates the overall architecture of the machine learning (ML) generative pipeline. The ML model is composed of (i) a forward validation module (consists of a curve type classifier and five individual surrogate neural networks) that predicts curve feature $\{\mathbf{X}^{P}\}$ given the design candidate $\{\mathbf{Y}\}$ and (ii) an inverse prediction module (consists of five individual generative neural networks) that predicts the design candidate $\{\mathbf{Y}\}$ based on the target curve feature $\{\mathbf{X}^{T}\}$. Each generative neural network is linked to its corresponding surrogate neural network, forming a

pair of generative-surrogate model. In total, we have five pairs of generative-surrogate model in our ML pipeline (Supplementary Fig. 1b).

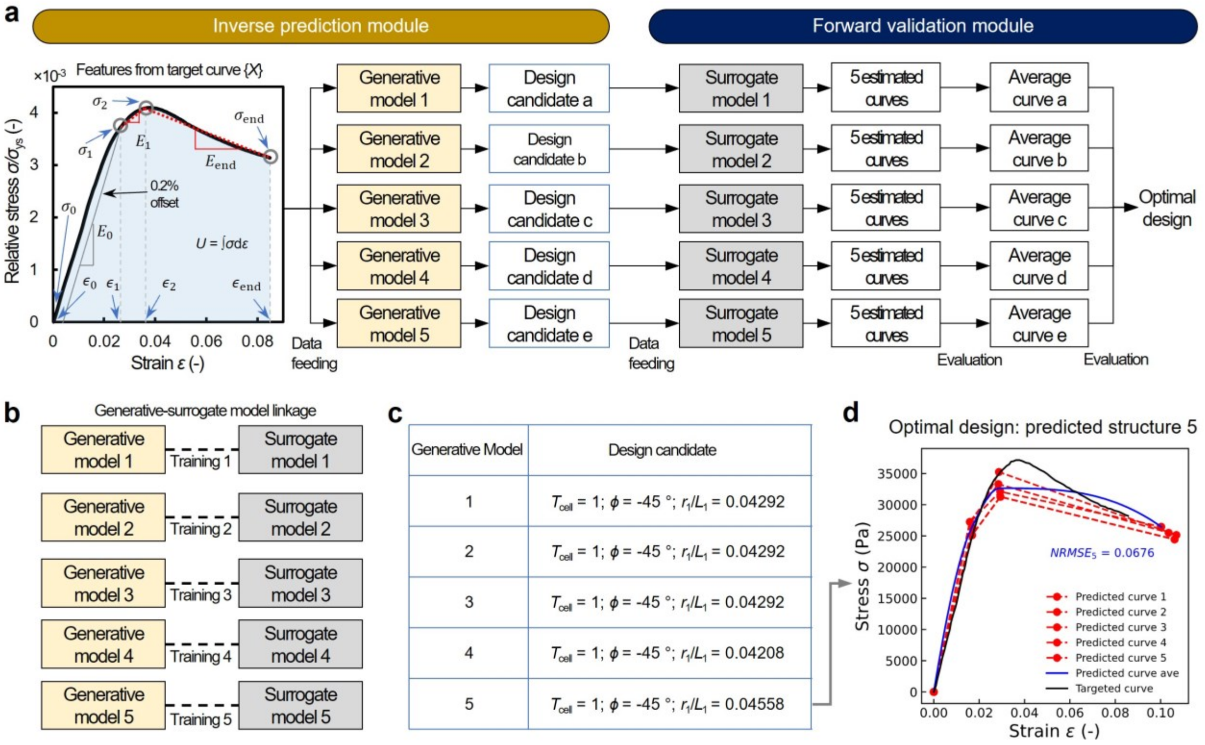

Supplementary Fig. 1 | Details of ML approach. a, The generative ML pipeline consists of two modules: inverse prediction module and forward validation module. Taken the target stress-strain curve feature as the input, the inverse prediction module predicts five design candidates, which are then passed to the forward validation module to evaluate the mechanical responses and select the optimal design. **b**, Five pairs of generative neural networks and surrogate neural networks in the ML pipeline. **c**, Multiple design candidates obtained from the inverse prediction module for the target curve shown in **a**. **d**, Example of estimated curves from the surrogate model 5. These curves were averaged and compared with the target curve in terms of the normalized root-mean-square error (NRMSE). After repeating this process for all models, the optimal design paired with a curve

exhibiting the minimum NRMSE was chosen. When the minimum NRMSE values are identical across multiple designs, the design candidate from a ML model with the highest prediction accuracy is chosen as the optimal design.

Although this model adopts generative neural networks that aim to predict an accurate lattice architecture {**Y**} for a given input target curve feature {$\mathbf{X}^T$}, our ML pipeline does not rely on any ill-defined cost function. Indeed, instead of defining the cost function based on a comparison between the predicted and true lattice architecture, the predicted lattice architecture {**Y**} is now converted back to a predicted stress-strain curve via the pretrained forward validation module (Sections 7.1-7.2). This modification allowed us to directly evaluate the difference between the predicted curve feature {$\mathbf{X}^P$} and the target curve feature {$\mathbf{X}^T$} via a new cost function $f_c=(\mathbf{X}_{pred}-\mathbf{X}_{true})^2$. As the stress-strain curve is unique to a lattice architecture, this approach effectively ensures the uniqueness of the solution and eliminates the "one-to-many" issue without compromising the properly defined nature of the cost function.

After predicting the curve features {$\mathbf{X}^P$} by the surrogate neural network model, the associated curve can be reconstructed based on the curve features. Each curve is uniquely described by a series of control points, which can be classified into five categories: origin, linear elastic limit, local maximum points, local minimum points, and end point (Supplementary Fig. 2a). In turn, a linear connection of these control points can approximately represent the true stress-strain curve, as illustrated in Supplementary Fig. 2b. The linear connection is called feature-to-curve reconstruction, and this linearly connected curve is herein referred as a reconstructed curve for the

curve features $\{\mathbf{X}\}$. We found that, when compared to the ground-truth stress-strain curves, the reconstructed curves exhibit very small discrepancy, with an average normalized root-mean-square error (NRMSE) of ~0.01; that is, the reconstructed curves largely overlap with the ground-truth curves. Supplementary Fig. 2c presents two representative cases of true curve and reconstructed curve. Herein, NRMSE is defined as:

$$\mathrm{NRMSE} = \sqrt{\frac{\sum_{i}^{46}\left(\overrightarrow{x^{i}}_{\mathrm{pred}}/\overrightarrow{x^{i}}_{\mathrm{target}} - 1\right)^{2}}{N_{\mathrm{features}}}} \quad (1)$$

where $\overrightarrow{x^{i}}_{\mathrm{pred}}$ and $\overrightarrow{x^{i}}_{\mathrm{target}}$ are i-th curve feature of $\{\mathbf{X}^{\mathrm{P}}\}$ and $\{\mathbf{X}^{\mathrm{T}}\}$, respectively, and N_{features} denotes the number of the curve features. This expression leads to the computed NRMSE bounded between 0 and 1, where 0 implies the two vectors are identical with 1 implying that they are completely dissimilar.

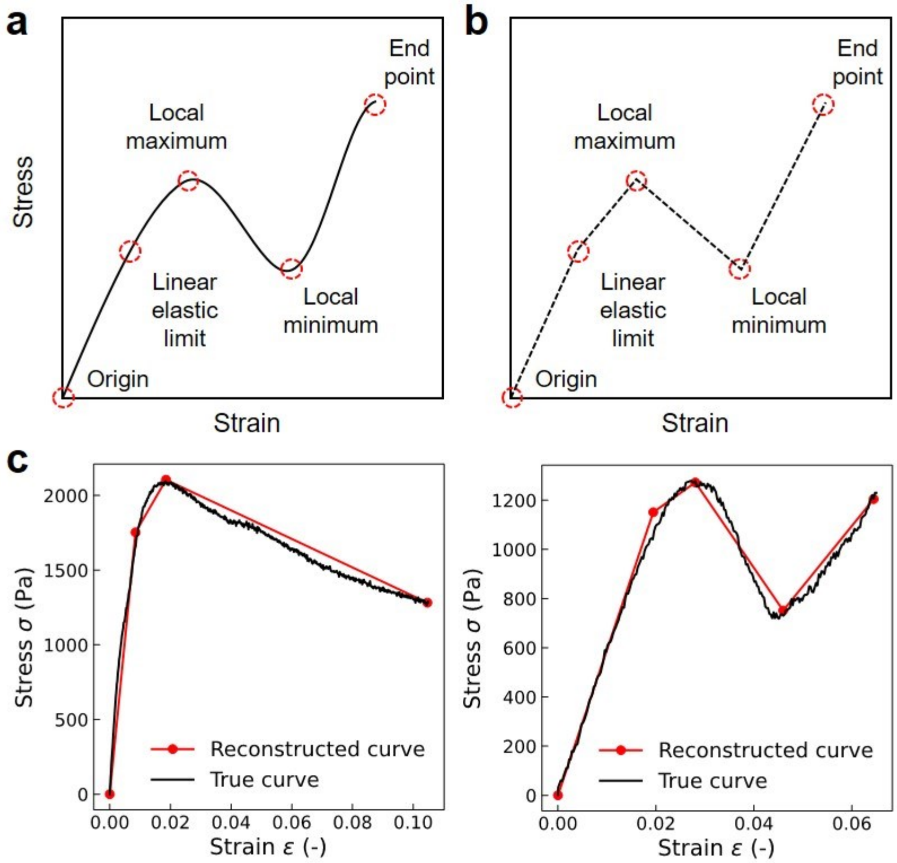

Supplementary Fig. 2 | Stress-strain curve reconstruction based on curve features. **a**, The original stress-strain curve with five types of control points. **b**, Reconstructed stress-strain curve via linear connection of all the control points. **c**, Two representative cases of comparison between true curve and reconstructed curve.

Note that not every curve contains all the five categories of control points. Along with the prediction of the curve features $\{\mathbf{X}^{\mathrm{P}}\}$, we also used the curve type classifier to predict the curve type (Supplementary Note 1.3). Based on the curve type, we were able to filter out the nonzero predictions of some curve features that are expected to be zero.

Supplementary Note 1.2. Determination of the optimal design candidate

We now discuss the strategy that was used herein to determine the optimal design from several candidate structures offered by multiple generative models (i.e., a series of independent generative models that are trained on the same dataset, but with different random initial weights and distinct hyperparameters). In detail, by inputting the same target stress-strain curve feature, each generative model independently predicts a design candidate, which might be different from each other. Although all the generative models may exhibit satisfactory accuracy, some predicted structure can provide a stress-train curve closer to the target curve than the other design candidates, which is the optimal design that should be picked out.

Supplementary Fig. 1a illustrates the model evaluation strategy to determine the optimal design. The inverse prediction module comprises five independent generative neural network models, wherein each one predicts its own design candidate $\{\mathbf{Y}\}$ for the given target curve feature $\{\mathbf{X}^{\mathrm{T}}\}$. These design candidates are then passed to the corresponding surrogate forward model to evaluate its response $\{\mathbf{X}^{\mathrm{P}}\}$ (Supplementary Fig. 1b). Each surrogate model predicts five sets of stress-strain curve feature for the input design candidate, and these five predicted curve features are then averaged to obtain the averaged stress-strain curve feature. In total, five surrogate forward model predicts five individual averaged stress-strain curve features, which are then compared with the input curve feature $\{\mathbf{X}^{\mathrm{T}}\}$ for the selection of optimal design $\{\mathbf{Y}\}$. The optimal design is then determined as the one that yields the smallest NRSME value compared with the input curve feature $\{\mathbf{X}^{\mathrm{T}}\}$. Supplementary Figs. 1c-d present a representative case of optimal design selection. For a case where the minimum NRMSE values are identical between multiple predicted curves, the

design candidate from a neural network model with the minimal training loss value is selected as the optimal design (detailed loss value for each neural network is given in Supplementary Fig. 17). Details of the design candidate evaluation are described in Supplementary Information section 7.5.

Supplementary Note 1.3. Curve type classifier

Given one set of design parameters $\{\mathbf{Y}\}$ and load type T_{load}, we first aimed to build a forward model that can predict the corresponding stress-strain curve $\{\mathbf{X}^{\mathrm{P}}\}$. In practice, there exists a discrepancy between the true curve $\{\mathbf{X}^{\mathrm{T}}\}$ and the predicted curve $\{\mathbf{X}^{\mathrm{P}}\}$. For example, in the case of a buckling response, $\{\mathbf{X}^{\mathrm{P}}\}$ generally exhibits a nonzero value (rather than a value of zero) for the local minimum stress and strain, which makes it difficult to differentiate with multiple peak-and-valley response.

To differentiate the curve type for each $\{\mathbf{X}^{\mathrm{P}}\}$, we herein constructed a curve type classifier using a neural network model implemented in the Python TensorFlow platform. The classifier contains 2 hidden dense layers with 64 and 128 neurons, respectively (Fig. 1a). We used the rectified linear unit (ReLu) activation function for the hidden layers and softmax activation for the output layer. Each hidden layer is followed by a batch normalization layer to improve the training efficiency (details of hyperparameters selection described in Supplementary Information section 7.1). By inputting the design parameter $\{\mathbf{Y}\}$ and load type T_{load}, the classifier outputs the probability of each curve type, i.e., linear, plastic yielding, buckling, and multiple peak-and-valley response (Supplementary Fig. 3).

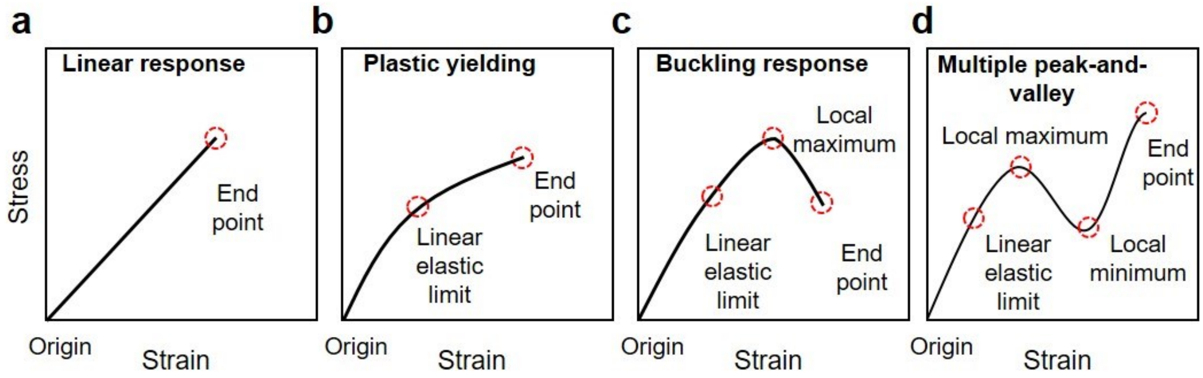

Supplementary Fig. 3 | Categories of stress-strain curves in the training dataset. a, Linear response. **b**, plastid yielding response. **c**, Buckling response. **d**, Multiple peak-and-valley response.

Supplementary Note 2. Derivation of stress-strain curve design space

In a dimensionless plot where the *x*-axis specifies the strain, and the *y*-axis specifies the relative compressive strength σ/σ_{ys} (σ_{ys} denotes the yield strength of the base material), each subdesign space, as illustrated in the inset of Fig. 2a, is constructed with three boundaries:

i. A lower boundary for the strain axis or *x*-axis (Ⓐ): described by the ratio of the maximum attainable strength to the highest attainable stiffness;

ii. An upper boundary for the relative compressive strength axis or *y*-axis (Ⓑ): represented by the maximum attainable strength;

iii. An upper boundary for the strain axis or *x*-axis (Ⓒ): characterized by failure strains and the corresponding maximum strengths.

The lower boundary for the strain axis Ⓐ can be determined by the maximum achievable stiffness and yield strength of an isotropic cellular material. These mechanical properties scale with the relative densities ($\bar{\rho}$) of the cellular materials[1]. Therefore, we estimate the maximum designable relative density ($\bar{\rho}_{max}$) as:

$$\bar{\rho}_{\max} \approx \sqrt{\frac{23}{12}\frac{\sigma_{\text{target}}}{\max(\sigma_{\text{ys}})}} \quad (2)$$

where σ_{target} is defined as the maximum strength of the target stress-strain curve and max(σ_{ys}) denotes the maximum yield strength of available base materials. Based on the maximum relative density ($\bar{\rho}_{max}$), the maximum achievable stiffness and yield strength are obtained using the Hashin-Shtrikman[2] and Suquet[3] bounds as:

$$\frac{E_{HSU}}{E_s} = \frac{2\bar{\rho}_{max}(5\nu - 7)}{13\bar{\rho}_{max} + 12\nu - 2\bar{\rho}_{max}\nu - 15\bar{\rho}_{max}\nu^2 + 15\nu^2 - 27} \tag{3}$$

$$\frac{\sigma_{y,SU}}{\sigma_{ys}} = \left(\frac{23}{12} - \frac{11}{12}\bar{\rho}_{max}\right)^{-1/2} \tag{4}$$

where the subscript s denotes the material properties of the base material. The lower boundary for the strain axis is marked as Ⓐ in the inset of Fig. 2a.

The upper boundary for the relative compressive strength axis Ⓑ is approximated as the theoretical upper bound of the yield strength (Supplementary Equation (4)) with $\bar{\rho}_{max}$ (inset of Fig. 2a). This treatment describes that failure occurs when local maximum stress within the lattice attains the yield strength of the solid constituent material.

The upper boundary for the strain axis Ⓒ is characterized by the maximum failure strain (ε_f) and the corresponding maximum strength ($\sigma_{y,SU}/\sigma_{ys}$) evaluated at designable relative densities ranging from 0 to $\bar{\rho}_{max}$ (inset of Fig. 2a). The estimated failure strain is given as:

$$\varepsilon_f = \left(\frac{\sigma_{ys}}{E_s}\right)\frac{1}{\bar{\rho}^{1/2}} \tag{5}$$

and the corresponding maximum strength is given in Supplementary Equation (4). The failure strain (ε_f) follows a power function of base material properties (σ_{ys} and E_s) and reaches the elastic limit (ε_{ys}) of the base material when $\bar{\rho} = 1$. This function reflects a general deformation trend of architected materials—low-density materials fail at a higher strain than high-density materials while the maximum strength at failure decreases gradually.

A representative subdesign space, constructed by the boundaries described above, is illustrated in the inset of Fig. 2a. The full stress-strain curve design space is formulated by superimposing a series of subdesign spaces based on all available base materials, truncated by densification (the black dotted region in Fig. 2a). Densification[1] is estimated as ε_d=1-1.4$\bar{\rho}$, where $\bar{\rho}$ varies from 0 to $\bar{\rho}_{max}$. This representation not only account for base material dependency on the stress-strain curves but also realize the broadest possible design space, owing to the subdesign space boundaries evolving with the base material properties (the gray curves in Fig. 2a).

Supplementary Note 3. Plottable stress-strain curve paths

Linear segment: The first segment (linear-elastic segment) of the target stress-strain curve starts with a straight-line (Supplementary Fig. 4a). This line is defined by two control points—the first control point (ε_0, σ_0) is located at the origin, and the second control point (ε_1, σ_1) can be anywhere within the design space. Two representative straight lines are shown in this figure. The upper limit of the slope for this line is given by the theoretical upper limit of the elastic stiffness (Supplementary Equation (3)), whereas the maximum value of σ_1 is defined by the theoretical upper limit of the yield strength (Supplementary Equation (4)), as described in the previous section.

Nonlinear segment: The following segment of the target curve accompanies with peaks and valleys (Supplementary Figs. 4b-c). The maximum number of peaks and valleys ($\max(N_{\mathrm{pv}})$) of the curve depends on which subdesign space of the first linear-elastic segment is contained within. Within the subdesign space, multiple peaks and valleys may exist when the slope of the first linear-elastic segment is low enough to trigger elastic instability (i.e., satisfying $\sigma_1/\varepsilon_1<A(\varepsilon_{\mathrm{ys}})^{\alpha}$, where A and α are lattice topology (strut orientation) dependent coefficients). The maximum number of achievable peaks and valleys ($\max(N_{\mathrm{pv}})$) is determined by print volume (L^3) and minimal printable feature size (s_{min}) that could experience elastic instability compared to the overall volume of the sample to be designed (i.e., $\max(N_{\mathrm{pv}})=B(L/s_{\mathrm{min}})(\sigma_1/\varepsilon_1)^{\beta}$, where B and β are lattice topology dependent coefficients).

The possible ranges and bounds of the peaks and valleys (yellow shaded region) are specified as shown in Fig. 2b. The location of the peaks and valleys are determined by the tangent modulus and displacement of the sample from the previous peak. The tangent slope between the subsequent peaks and valleys is lower or equal to the linear-elastic slope before first yielding is reached (i.e., $|(d\sigma_{\mathrm{pv}}^{i})/(d\varepsilon^{i})|\leq(d\sigma_{\mathrm{y}}^{1})/(d\varepsilon^{1})$, where i = 2,…, max(N_{pv})). The x-coordinate (the strain axis) of the peaks and valleys corresponds to the already collapsed unit cell sections (i.e., $\Delta\varepsilon=(s_{\mathrm{min}}/L)C^{\gamma}\,(\sigma_{1}/\varepsilon_{1})^{\tau}$, where C, γ, and τ are lattice topology dependent coefficients). Then, the y-coordinate is determined by the tangent slope and the x-coordinate of the peaks and valleys.

Other curve features include: the y-coordinate of the last control point (σ_N) is larger than the previous value (i.e., $\sigma_N > \sigma_{N-1}$), and that no discontinuity shall be presented in the curve that should be differentiable at any control points (i.e., $\lim_{x\to x^-}\frac{dy}{dx} = \lim_{x\to x^+}\frac{dy}{dx}$).

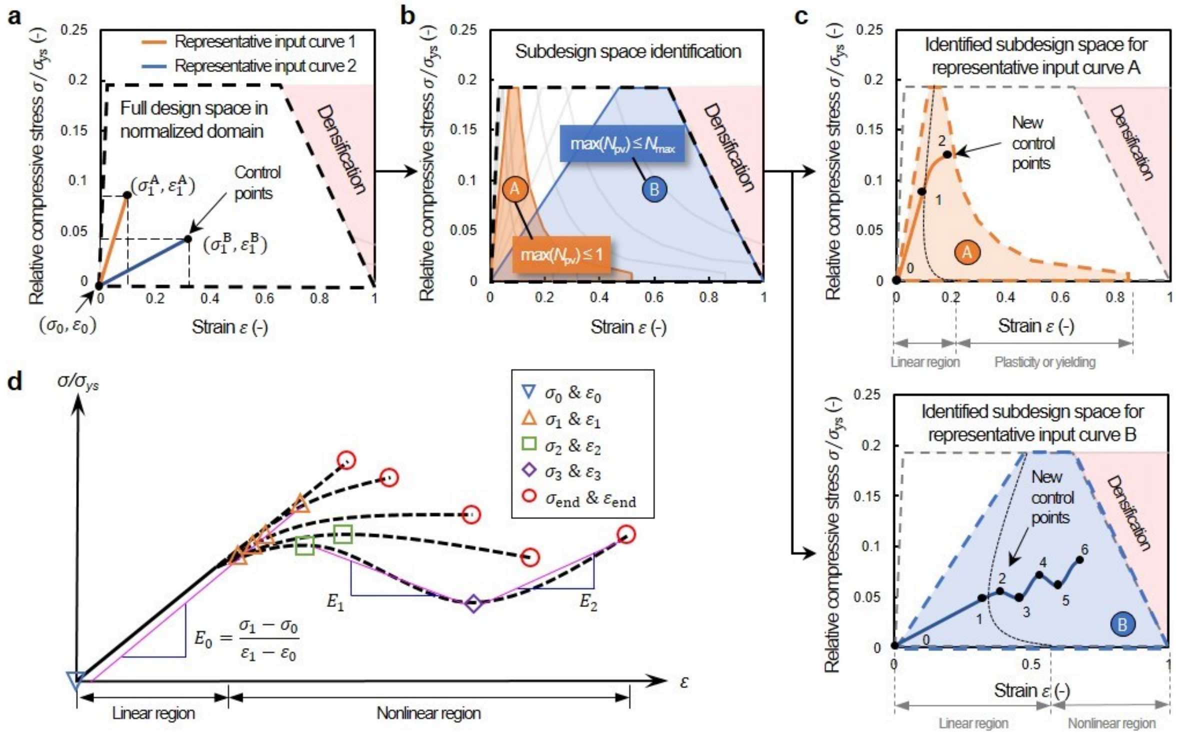

Supplementary Fig. 4 | Plottable stress-strain curve paths. **a**, Linear segments of two representative target curves in the full design space from user input. **b**, Contour map showing the achievable number of peaks and valleys of the curves based on the subdesign space. **c**, Nonlinear segments of the representative target curves within the corresponding subdesign space, which completes a sketch of the curves. **d**, Example of compressive stress-strain curve paths described by several curve features for a special case of max(N_{pv}) equal to unity.

Supplementary Note 4. Stress-strain curve parameterization

Stress-strain curves were parameterized by curve features (X_i), where i ranges from 1 to 6×max(N_{pv})+10 and max(N_{pv}) describes the maximum number of achievable peaks and valleys (see details of plottable stress-strain curve paths in Supplementary Note 3). These curve features were specified by identifying control points (ε_j, σ_j) from the curve where j varies from 0 to 2×max(N_{pv})+2. A complete relationship between the curve features and control points are provided in Supplementary Table 1. The stress-strain curve parameterization process was implemented in Python with numpy[4], SciPy[5], and pandas[6] packages. The following sections describe the parameterization process in case of max(N_{pv}) equal to 6 in details.

In case of the training curve, the first step of the parameterization process was an identification of their control points (Supplementary Fig. 5a). The identified control points were then assigned to the curve features according to the descriptions listed in Supplementary Table 1. In detail, the identification process begins with filtering noisy data presented in the curve to minimize any data fluctuations and inconsistency[7] by using the Savitzky-Golay definition[8]. Upon the completion of the filtering process, the beginning of the curve was set to (ε_0, σ_0), and a load type (T_{load}) was determined by recognizing whether the initial and terminating stress values of the curve were identical. A non-linear segment at the beginning of the curve (also called as the toe-in region) was also detected and temporarily deactivated to minimize inaccuracy in the following elastic modulus (E_0) measurement (i.e., the slope of the linear segment of the curve). The elastic modulus was computed by using a linear least-squares regression[5] with the coefficient of determination set to 0.999. According to a typical 0.02% strain offset method, a straight line with a slope described by the computed E_0 was determined, from which an intersecting point of this line and the curve was

set to (ε_1, σ_1), representing the termination of linearity. When this point was not detectable, the end point of the curve was set to (ε_{end}, σ_{end}) which represents failure without appreciable yielding as illustrated in the upper left sub-figure in Supplementary Fig. 5b. Subsequent stresses and strains after the linear segment—control points denoted by (ε_j, σ_j) where j = 2…13—were identified by locating local maxima and minima of the curve (using signal.find_peaks and signal.argrelmin of the SciPy package[5]). Finally, (ε_{end}, σ_{end}) were set by recognizing terminating stress and strain values of the curve. Once all control points were identified, they were assigned to the corresponding curve features (X_i where i = 5…32) according to the control point-curve feature relationship provided in Supplementary Table 1.

The remaining curve features (X_i where i = 1…4 and 33…46) were recognized from the previously determined T_{load} and E_0 as well as additional variables such as strain energies and tangent moduli in the nonlinear segment of the curve. Tangent moduli ($E_1 \sim E_{13}$) were determined by computing a slope between two adjacent stress and strain values (i.e., $E_i = (\sigma_{j+1} - \sigma_j)/(\varepsilon_{j+1} - \varepsilon_j)$). In addition, stored and released strain energy per unit volume (denoted by U^{loading} and $U^{\text{unloading}}$, respectively) were computed by a trapezoidal integration method (e.g., $U^{\text{loading}} = \int_{\varepsilon_0}^{\varepsilon_{\text{end}}} \sigma d\varepsilon$) using numpy.trapz of the numpy package[4], and a dissipated strain energy (ΔU) was obtained by subtracting $U^{\text{unloading}}$ from U^{loading} (i.e., $\Delta U = U^{\text{loading}} - U^{\text{unloading}}$). As before, these variables were assigned to the corresponding curve features as listed in Supplementary Table 1, and this completed the stress-strain curve parameterization in case of the training curve.

In case of the target curve, the aforementioned identification process of the control points was not necessary as these points were to be specified by the user, as discussed in the main text and

Supplementary Note 3. Hence, with the user-specified control points, the curve parameterization process was simply done by determining the additional variables discussed above and assigning them to the corresponding curve features, as listed in Supplementary Table 1.

Supplementary Table 1 | Feature variables parameterizing the stress-strain curve in case of $\max(N_{\mathrm{pv}}) = 6$.

Curve feature {X}	Description	Method of determination
X_1	Loading type (T_{load})	0 (i.e., monotonic response) if $\sigma(\varepsilon_0) \neq \sigma(\varepsilon_{\mathrm{end}})$<br>1 (i.e., cyclic response) if $\sigma(\varepsilon_0) = \sigma(\varepsilon_{\mathrm{end}})$
X_2	Stored energy per unit volume (U^{loading})	$U^{\mathrm{loading}} = \int_{\epsilon_0}^{\epsilon_{\mathrm{end}}} \sigma^{\mathrm{loading}} d\epsilon$
X_3	Released energy per unit volume ($U^{\mathrm{unloading}}$)	$U^{\mathrm{unloading}} = \int_{\epsilon_0}^{\epsilon_{\mathrm{end}}} \sigma^{\mathrm{unloading}} d\epsilon$
X_4	Dissipated energy per unit volume (ΔU)	$\Delta U = U^{\mathrm{loading}} - U^{\mathrm{unloading}}$
X_5 , X_7, … X_{29}	Stress (σ_i)	Note: 0.2% offset method used for $i = 1$
X_6, X_8, … X_{30}	Strain (ϵ_i)	
X_{31}	End stress (σ_{end})	End of the curve if $T_{\mathrm{load}} = 0$<br>Maximum strain and the corresponding stress if $T_{\mathrm{load}} = 1$
X_{32}	End strain (ϵ_{end})	
$X_{33} \sim X_{46}$	Stiffness (E_i)	$E_i = \frac{\sigma_{i+1}-\sigma_i}{\epsilon_{i+1}-\epsilon_i}$ where $j = 1 \ldots 2\times\max(N_{\mathrm{pv}})+2$<br>Note: elastic stiffness when $i = 0$

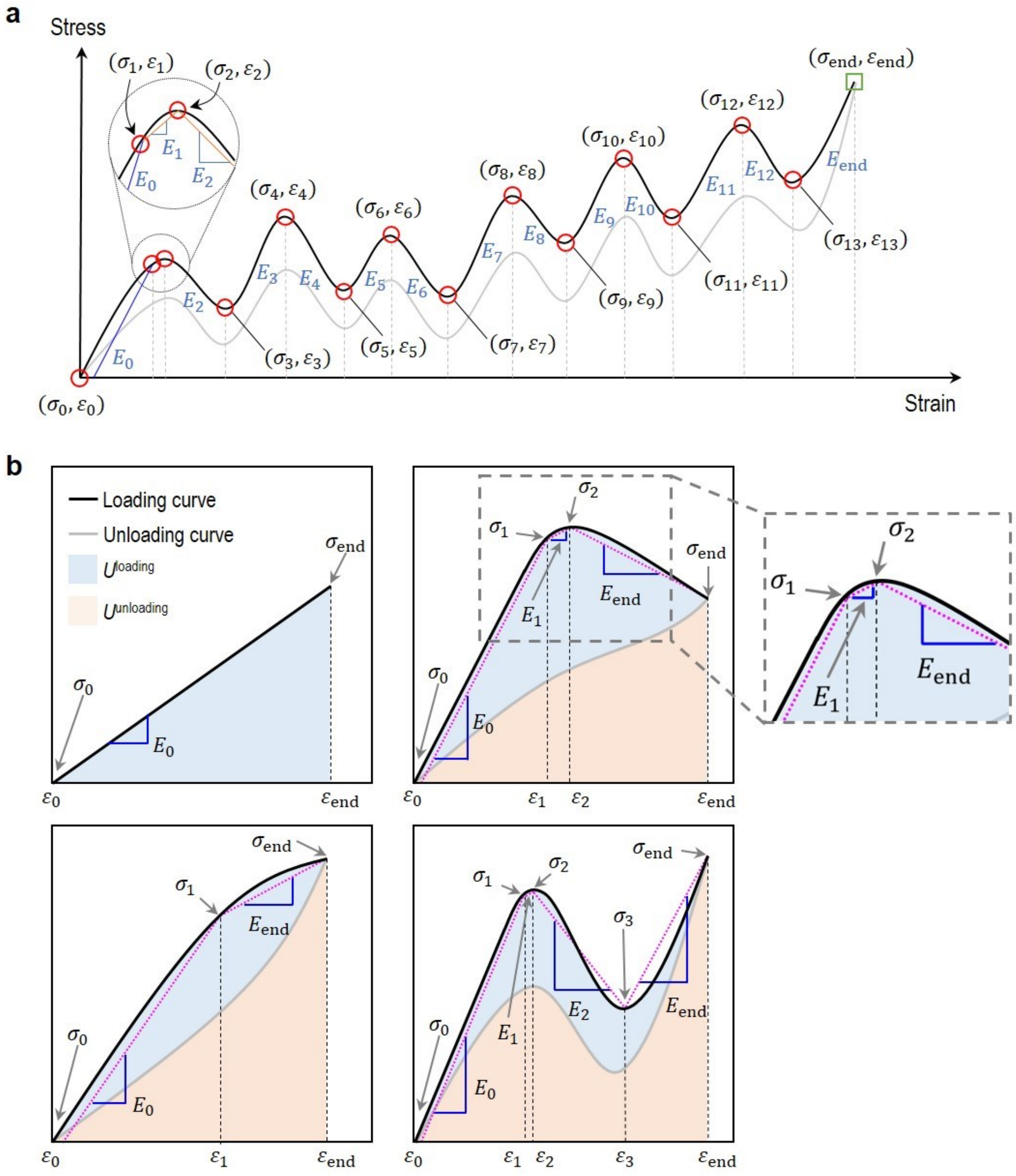

Supplementary Fig. 5 | Curve parameterization used in this work. a, Stress-strain curve parameterized in terms of curve features to fully describe its important mechanical properties. For clarity, a case of $\max(N_{pv}) = 6$ is illustrated, and the variables describing the loading type and energy terms (i.e., T_{load}, $U^{loading}$, $U^{unloading}$, ΔU) are not shown. **b,** Examples of the curve parameterization for several curve paths.

Supplementary Note 5. Choice of architectural unit cells

We conducted FE simulations to compare plate-based (BCC, FCC, and SC-FCC) and open-cellular lattices (the five cell types used in this study) at the relative density ($\overline{\rho}$) ranging from 5 to 25% in representative subdesign spaces (Supplementary Fig. 6). The stress-strain curve results show that plate-base lattices (i.e., BCC, FCC, and SC-FCC) cover narrow (green-shaded) regions indicated in these figures and are limited to a linear-elastic response followed by failure due to yielding (Supplementary Fig. 6a). The curve shapes are owing to their highly connected edges between plate members (rather than node connectivity)—their limited stress-strain curves were revealed in recent studies[9,10]. The open cellular lattices, when modeled with the same range of the relative densities, exhibit more diverse stress-strain curves and broader coverage in both subdesign spaces than those of the plate-lattices (light-blue shaded regions in Supplementary Figs. 6b-c; representative curves in Supplementary Fig. 6b). While the plate-based lattice covers the upper left corner on the design space (approaching the theoretical upper bound), such a region can be achieved by open cellular lattices made with higher relative densities (highlighted by blue-shaded regions in Supplementary Figs. 6b-c). We note that these findings are also valid for other base materials, given that the two subdesign spaces represent relatively extreme cases of available base materials.

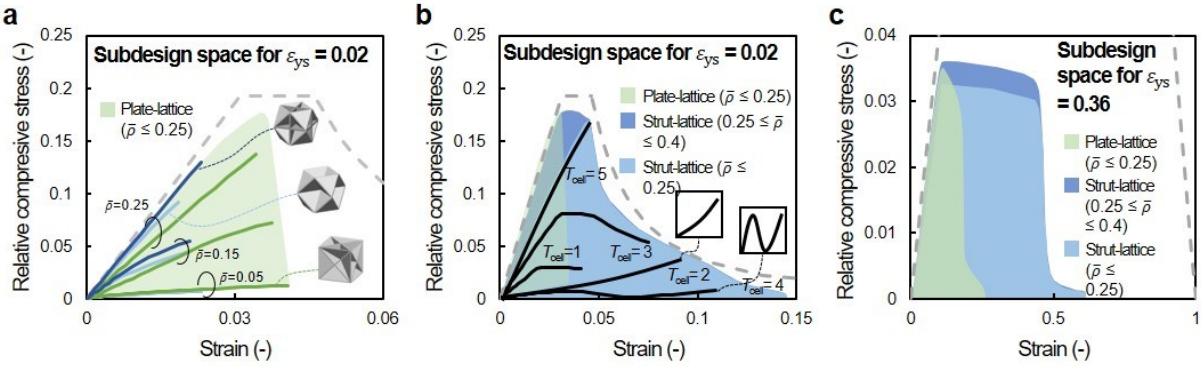

Supplementary Fig. 6 | Mechanical performance assessment of the architectural unit cells developed in this work. a, Results of FE simulations showing representative stress-strain curves of typical plate-lattices in a subdesign space for ε_{ys} of 0.02 (relatively brittle base material). In this figure, responses of SC-FCC, BCC, and FCC are colored as blue, light blue, and green, respectively. **b**, The results of FE simulations showing representative stress-strain curves of the strut-based architectural unit cells (T_{cell} of 1 through 5) and their coverages in the same subdesign space for a comparison. **c**, The results of FE simulations illustrating coverages of the strut-lattices and plate-lattices in another representative subdesign space for ε_{ys} of 0.36 (relatively flexible base material).

FE simulations were performed on lattices made of the proposed architectural cells to study their size effect. Periodic lattices for each cell were modeled with identical overall dimensions with the number of unit cells per side (N_{cell}) from 2 to 10. In compound lattices, a 3×3×3 compound lattice configuration was treated as the smallest repeating geometry (N) and was tessellated in three orthogonal directions. We started from a compound lattice having one design gradient (G_1: cell type), and other design gradients (G_2: strut radius ratio, G_3: inclined strut radius, and G_4: cell size) were consecutively added to the previous configuration one-by-one. Other settings such as

constituent material modeling and loading and boundary conditions can be found in Methods – Finite element simulation.

FE results show that both the elastic stiffness and peak strength have minimal size effects regardless of the cell type and lattice uniformity (Supplementary Figs. 7-8). These findings align well with the previous study[11-13].

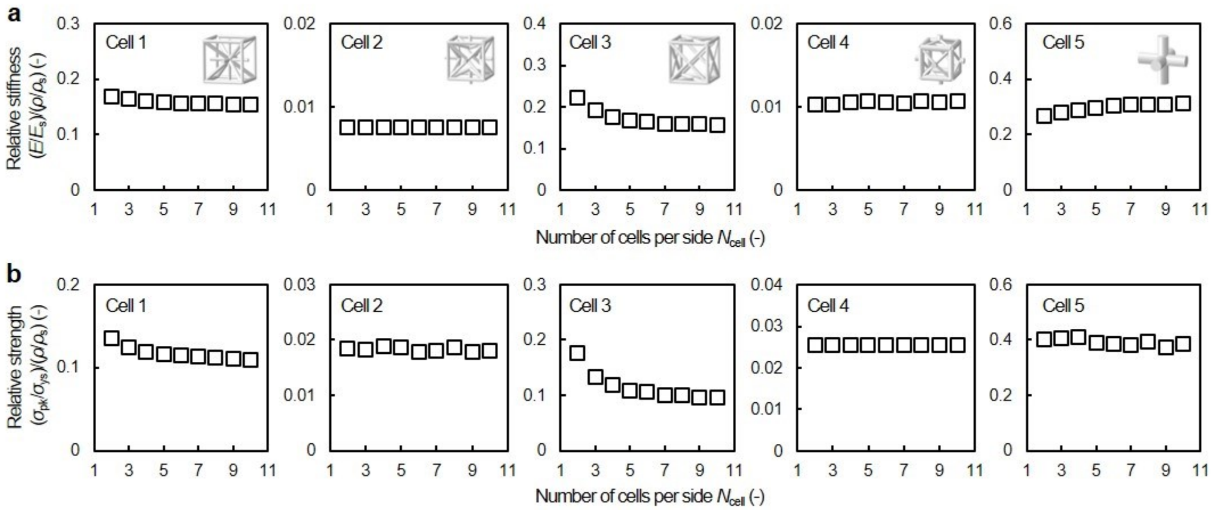

Supplementary Fig. 7 | FE results showing size effects of the periodic lattices made of the presented architectural cells as a function of the number of unit cells (N_{cell}). a, Relative compressive stiffness. **b**, Relative compressive strength.

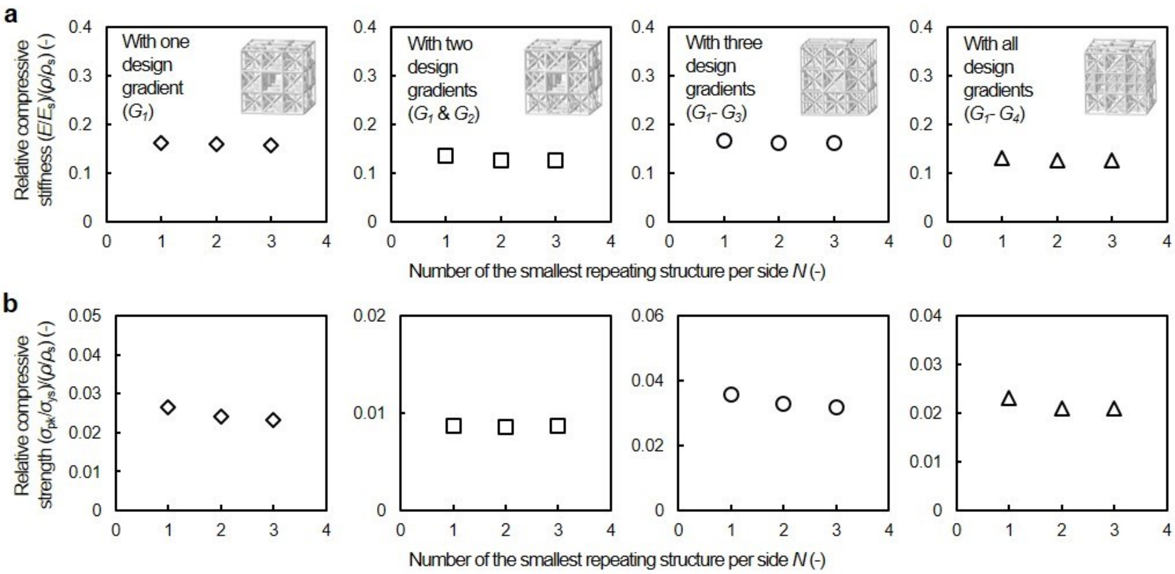

Supplementary Fig. 8 | FE results showing size effects of the compound lattices as a function of the number of the smallest repeating geometry (*N*). **a**, Relative compressive stiffness. **b**, Relative compressive strength. Each design gradient (G_1: cell type, G_2: strut radius ratio, G_3: inclined strut radius, and G_4: cell size) were consecutively added to the previous configuration one-by-one.

Supplementary Note 6. Generation of training instances

To generate training instances on the basis of the developed architectural cells (Fig. 2c), we discretized the characteristic angle (ϕ) (i.e., the projected inclined strut onto the 13- or 23-plane) into 18 discrete values ranging from -45 to 90°. More specifically, the angle for the architectural cell 2 was uniformly distributed between -30 and -6° with an interval of 4° to fill the gap in the ϕ domain between the architecture cells 1 and 3. Similarly, the angle for the architectural cell 4 was evenly spaced by 8 intervals from 6 to 34° to fill the gap in the ϕ domain between the architectural cells 3 and 5. The angles for the architectural cells 1, 3, and 5 were naturally prefixed to -45, 0, and 90° by design, respectively. We intentionally excluded some ϕ near cell boundaries (i.e., ϕ ranging between -30 and -45° for the architecture cell 2 and between 34 and 90° for the architecture cell 4) to distinctly differentiate stress-strain curves of neighboring architectural cells while avoiding possible overlaps.

Next, for each discretized characteristic angle ϕ, we discretized the radius-to-length ratio of the inclined strut (r_1/L_1) into approximately 17 intervals. This discretization resulted in hundreds of architectural configurations with a range of the relative density ($\bar{\rho}$) spanning from ~3 to ~25% (Supplementary Table 2). The rationale of this relative density range is that, at higher relative densities, tuning stress-strain curves is limited as the curves become self-similar regardless of architectural cell types—they converge to those of a solid. This is attributed to an increased nodal volume which triggers failure at the node by plastic yielding and stress concentration rather than exploiting deformation mechanisms such as stretching, bending, and buckling of struts.

Following this, each architectural configuration was tessellated in three orthogonal directions to create a 3D digital model and was fabricated through additive manufacturing (see Methods – Sample fabrication). The printed samples were tested with two different loading conditions: uniaxial monotonic compression prior to failure and cyclic compression with three different maximum strains (measured stress-strain curves in Supplementary Fig. 9). Each measured stress-strain curve was then parameterized into 46 curve features {**X**} and paired with its corresponding design parameters {**Y**} (T_{cell}, ϕ and r_1/L_1) forming the pristine training dataset, containing around a thousand {**X**}-{**Y**} pairs.

Here, in the pristine training dataset, we adopted the one-hot representation of T_{cell} and normalized ϕ and r_1/L_1 to improve the training efficiency. The cell type is represented using a 1x5 vector as: cell 1 – [1,0,0,0,0] and cell 4 – [0,0,0,1,0]. While cells 1, 3 and 5 have a fixed value of ϕ, cells 2 and 4 have a fixed range of ϕ, we normalized the characteristic angle ϕ a fixed range of (0,1), where 0 and 1 represent the lower and upper limit of the angle respectively. The radius-to-length ratio r_1/L_1 is standardized to a normal distribution with a zero mean and a variance equal to 1.

Next, we performed data augmentation based on the pristine dataset to account for prediction fluctuation and achieve satisfactory prediction accuracy in the training of surrogate neural network models, which is "many-to one" mapping. This was achieved by adopting a standard SMOTE oversampling approach[14]. For cell types 1, 3 and 5, which characteristic angle ϕ is fixed, any predicted ϕ value between 0 to 1 shall represent the same ϕ value as the original fixed angle. Thus,

for cell types 1, 3, and 5, we augmented the dataset by generating new datasets with new varying normalized ϕ values between 0 and 1 (here, we select 0, 0.25, 0.5, 0.75, and 1). Moreover, since the inverse prediction of the one-hot-encoded T_{cell} is performed by using a "softmax" activation function, the predicted one-hot element would be close to, but less than 1, e.g., 0.95. To account for the prediction fluctuation in the one-hot-encoded T_{cell} feature, we further augmented the dataset by generating new data points with various new one-hot T_{cell} values—herein, we generate four new one-hot-encoded T_{cell} values for each datapoint, i.e., each $\{\mathbf{X}\}$-$\{\mathbf{Y}\}$ pair. In detail, the one-hot-encoded element was randomly selected from a uniform distribution between 0.9 and 1.0, and the remaining non-hot-encoded elements were also randomly selected from a uniform distribution between 0.0 and 1.0, with a prerequisite that the sum of all elements in each one-hot representation is equal to 1, e.g., [0.95, 0.01, 0.02, 0.005, 0.015] for cell type 1. After the data augmentation, the dataset contains approximate ten thousand $\{\mathbf{X}\}$-$\{\mathbf{Y}\}$ pairs.

Each stress-strain curve is uniquely then described by 46 curve features $\{\mathbf{X}\}$, as previously described in Supplementary Note 4. During training, we standardized these 46 curve features to improve the training efficiency. Considering the fact that the strain and stress values range over several orders of magnitude, we transformed each curve feature into a logarithmic representation prior to standardization so as to reduce their standard deviation. Note that some of the curve features does not exist in the curve for partial training dataset. For instance, some curves may not exhibit any local minimum or maximum. In such cases, we simply set the relevant feature values to zero.

However, we found that the zero representation of nonexistent curve features herein turns out to be very challenging to be accurately predicted and, in addition, negatively affectively affect the prediction of existent features in the training process. This situation is likely to arise from the giant gap of magnitude between the zero value and the existent curve features. To address the "zero-type" issue, the zero value for each nonexistent feature was replaced by a relevant curve feature with nonzero representation. Specifically, in case of a monotonic curve, ΔU was set as the area under the curve. Similarly, we reset the nonexistent strain and stress features to the values of their end strain ε_{end} and stress σ_{end} values, respectively. By doing so, the nonexistent features exhibit a magnitude that is similar to that of the existent features, and the nonexistent points are able to overlap with the end point to yield an identical stress-strain curve.

Based on the updated dataset, the load type T_{curve} is required to be added into $\{\mathbf{X}\}$ to identify the curve type associated with $\{\mathbf{X}\}$. Herein, we classified the stress-strain curves into 4 categories: linear response, plastic yielding response, buckling response and multiple peak-and-valley response. The curve type T_{curve} is encoded by one-hot representation, that is, [1,0,0,0], [0,1,0,0], [0,0,1,0], and [0,0,0,1] represent linear, plastic yielding, buckling, and multiple peak-and-valley response, respectively.

Supplementary Table 2 | Structure of training dataset.

Cell type T_{cell} (Y_1) (-)	Characteristic angle ϕ (Y_2) (deg)	Radius-to-length ratio of inclined strut r_1/L_1 (Y_3) (-)	Relative density ($\bar{\rho}$) (%)	Number of samples per loading condition	
				Monotonic compression	Cyclic compression
1	-45	0.027 ~ 0.069	2 ~ 15	19	57
2	-30	0.047 ~ 0.117	3.0 ~ 15	18	54
	-26	0.044 ~ 0.111	2.9 ~ 16	18	54
	-22	0.040 ~ 0.106	2.9 ~ 17	18	54
	-18	0.039 ~ 0.101	2.9 ~ 17	18	54
	-14	0.036 ~ 0.095	2.9 ~ 18	18	54
	-10	0.033 ~ 0.086	2.8 ~ 17	17	51
	-6	0.031 ~ 0.081	2.9 ~ 18	17	51
3	0	0.025 ~ 0.068	1.9 ~ 13	16	48
4	6	0.022 ~ 0.057	3.0 ~ 17	16	48
	10	0.027 ~ 0.084	2.9 ~ 26	16	48
	14	0.031 ~ 0.093	2.9 ~ 24	16	48
	18	0.035 ~ 0.103	3.0 ~ 23	16	48
	22	0.039 ~ 0.113	3.0 ~ 22	16	48
	26	0.044 ~ 0.123	3.0 ~ 20	16	48
	30	0.047 ~ 0.132	2.8 ~ 19	16	48
	34	0.052 ~ 0.172	2.8 ~ 25	16	48
5	90	0.112 ~ 0.292	2.8 ~ 17	16	48
Number of stress-strain curves per loading type				303	909
Total number of stress-strain curves				1212	

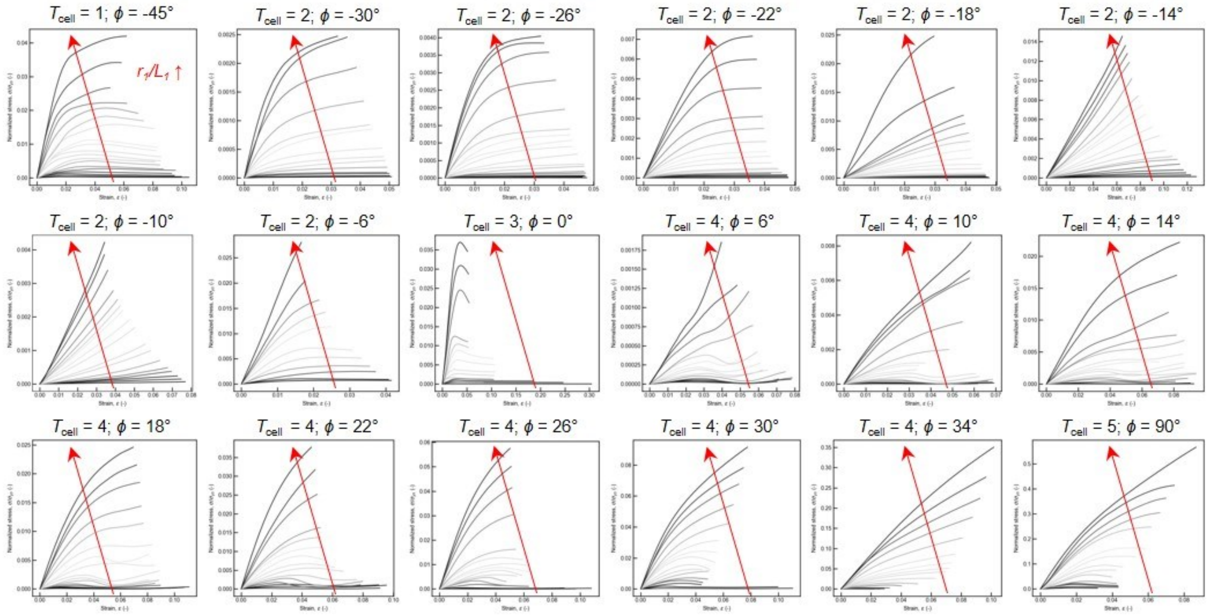

Supplementary Fig. 9 | Experimentally measured stress-strain curves of the as-printed lattice samples using the architectural cells developed in this study. For clarity, the curves under monotonic compression loading are shown. An architectural transformation from varying the characteristic angle (ϕ) facilitates a stress-strain curve evolution. Together with controlling the radius-to-length ratio of the inclined strut (r_1/L_1), each configuration exhibits a family of its own respective stress-strain curves.

Supplementary Note 7. Training of the machine learning

Supplementary Note 7.1. Training of the curve type classifier

We trained the classifier based on the augmented training dataset. Following a random train-test split 30% of the dataset was kept hidden to the model and used as test set later. We then adopted 10-fold cross-validation on the remaining 70% to adjust the hyperparameters (so as to minimize the cross-validation error). In detail, each batch contains 32 curves. The loss function L is defined as the mean square error between the predicted and targeted curve types. We adopted a stochastic gradient descent (SGD) optimizer to minimize the loss function with an initial learning rate of 0.01 and a Nesterov momentum of 0.9. The learning rate was set to decay one order of magnitude after a patience of 10 training epochs. Supplementary Fig. 10a shows the test set loss as a function of training epochs. After 100 training epochs, the test set loss converges to a miniscule level (~0.01).

We now investigate the classification accuracy of the curve type classifier. Supplementary Fig. 10b shows the confusion matrix of the classifier's predictions for the test set, where the test set contains 2808 curves in the augmented dataset. It is notable that the classifier can accurately predict the categories of all curve types, with the accuracy reaching ~98% for each curve type. Due to its high classification accuracy, the classifier can be used to filter nonzero predictions of curve features that are expected to be zero, thus facilitating the feature-to-curve reconstruction (Supplementary Note 1.1).

It should be pointed out that, we have optimized the classifier performance by tuning all relevant training hyperparameters (so as to maximize the cross-validation accuracy). For instance, Supplementary Fig. 10c shows the misclassification fraction of the test set as a function of the number of neurons in the first hidden layer. The number of neurons in the second hidden layer was herein set to be 2 times that of the first hidden layer, while other hyperparameters remain unchanged. We find that the classifier with a first hidden layer of $\geq$ 32 neurons can offer very accurate predictions of curve type, with a misclassification fraction lower than 1%. Hence, we selected 64 neurons in the first hidden layer to achieve an optimal balance between model accuracy and simplicity.

Moreover, we investigate the influence of the size of the training set on the classifier's accuracy, i.e., by constructing a learning curve. In that regard, Supplementary Fig. 10d shows the misclassification fraction (both the training and test sets) as a function of the size of training set, wherein five training sizes (i.e., 10%, 30%, 50%, 70%, 90% of the augmented dataset) were selected, and the dataset is randomly split into training and test sets. We found that, as expected, with more and more learning examples in the training set, the classifier exhibited an enhanced classification accuracy for the test set and then eventually reaches a plateau when the training size exceeds 50% of the dataset, with a classification accuracy of ~99% for both the training and test sets. These results confirmed that the model is not notably over- or under-fitted. We selected herein 70% of the dataset as training set to avoid the issue of sample deficiency that impairs the classifier performance.

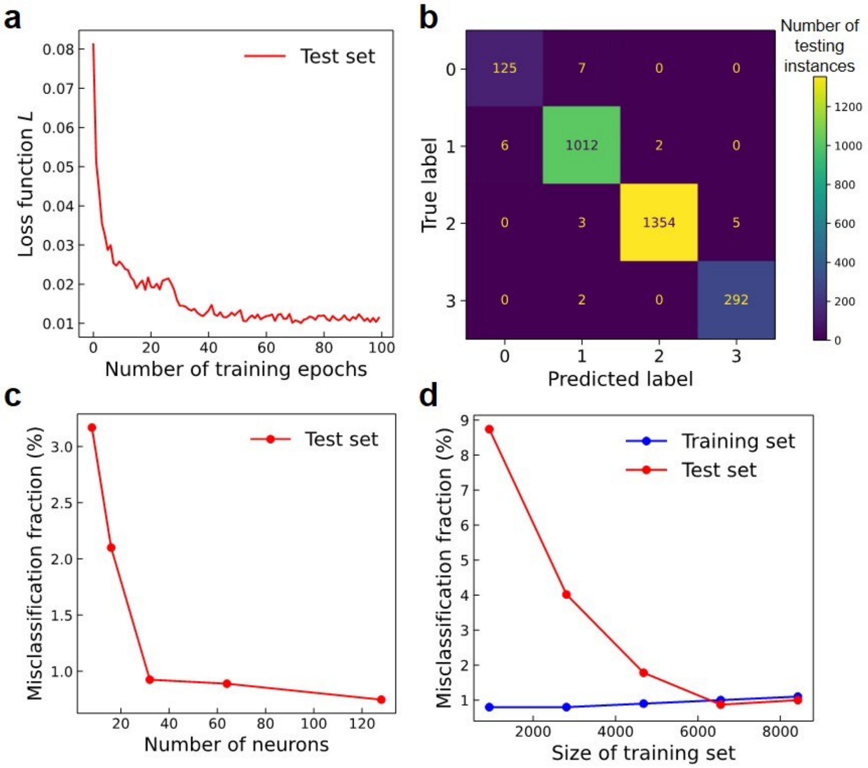

Supplementary Fig. 10 | Training results of the curve type classifier. a, Classifier loss L as a function of the number of training epochs for the test set. The test set contains 2808 curves and accounts for 30% of the dataset. **b**, Confusion matrix of the test set predicted by the curve type classifier. The label index ranging from 0 to 3 represents linear, plastic yielding, buckling, multiple peak-and-valley response, respectively. **c**, Misclassification fraction of the test set as a function of the number of neurons in the first hidden layer. Note that the number of neurons in the second hidden layer is set to be 2 times that of the first hidden layer. **d**, Misclassification fraction (for both the training and test sets) as a function of the size of the training set.

Supplementary Note 7.2. Training of the forward validation module

The forward validation module contains both a curve feature regressor (consists of five surrogate neural network models) and a pretrained curve type classifier. During training, the curve type

classifier was kept frozen with fixed hyperparameters. Similar to the training process of curve type classifier, we utilized the augmented dataset for the training of the forward validation module and first defined the test set comprising 30% of the data points by a random train-test split. We then trained the forward surrogate model (i.e., the curve feature regressor herein) using 10-fold cross-validation on the remaining 70% of the data points. In detail, each batch contains 32 curves. The loss function f_c is defined as the mean square error between predicted and targeted curve features, with the curve type being included. We adopted the stochastic gradient descent (SGD) optimizer to minimize the loss function with an initial learning rate of 0.01 and a Nesterov momentum of 0.9. The learning rate was set to decay 1 order of magnitude after a patience of 10 training epochs. Supplementary Fig. 11a shows the test set loss as a function of the number of training epochs. After 200 training epochs, the test set loss converges to a miniscule level (~0.05).

Note that, here, we have optimized the forward model performance by tuning all relevant training hyperparameters (to maximize the cross-validation accuracy). Supplementary Fig. 11b shows the final test loss as a function of the number of neurons in the first hidden layer in the curve feature regressor, wherein the number of neurons in the second hidden layer is twice as large as in the first hidden layer, while the other hyperparameters remain unchanged. As expected, larger number of neurons leads to smaller test loss, and we find that the test loss converges to a miniscule level when the first hidden layer contains $\geq$ 128 neurons. We selected herein 128 neurons in the first hidden layer, which offers an optimal balance between model accuracy and simplicity.

Next, we investigated the influence of the training size on the forward model performance by constructing a learning curve. Supplementary Fig. 11c shows the final loss as a function of the size of training set for both the training and test sets, wherein five training sizes (i.e., 10%, 30%, 50%, 70%, 90% of the dataset) are selected, and the dataset was randomly split into training and test sets. As expected, at small training size, the model exhibits a high test-loss due to the deficiency of training samples. Notably, with more and more learning examples in the training set, the forward model exhibits an enhanced prediction accuracy for the test set and then eventually reaches a plateau when the training size exceeds 50% of the dataset, with a miniscule final loss of ~0.06 for both the training and test sets. These results confirm that the model does not exhibit any notable level of over- or under-fitting. We selected herein 70% of the dataset as training set to avoid the issue of sample deficiency that impairs the forward model performance.

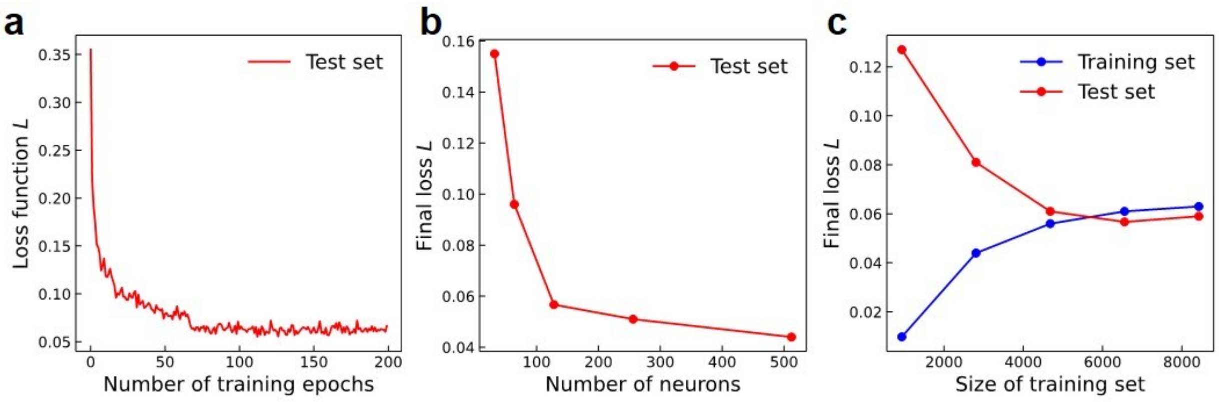

Supplementary Fig. 11 | Training results of the forward validation module. a, Forward prediction loss L as a function of the number of training epochs for the test set. The test set contains 2808 curves and accounts for 30% of the dataset. **b**, Final test loss as *L* as a function of the number of neurons in the first hidden layer. Note that the number of neurons in the second hidden layer is

set to be 2 times that of the first hidden layer. **c**, Final loss L (for the training and test sets) as a function of the size of training set.

Once trained, the forward neural network model acts as a surrogate model (that effectively replaces the simulations) that predicts the stress-strain curve of an input lattice design. To ensure that this model acts as an accurate surrogate simulator, we further investigated the prediction accuracy of the surrogate forward model. Supplementary Fig. 12 provides the predicted versus true values of come representative curve features offered by the forward surrogate model for the test set, where the test set contains 2808 curves. Note that the output curve features are standardized. For each feature, it is notable that all the datapoints are located at the vicinity of the $y = x$ identity line. These results demonstrate that the forward surrogate model can accurately predict all the curve features used to describe a stress-strain curve.

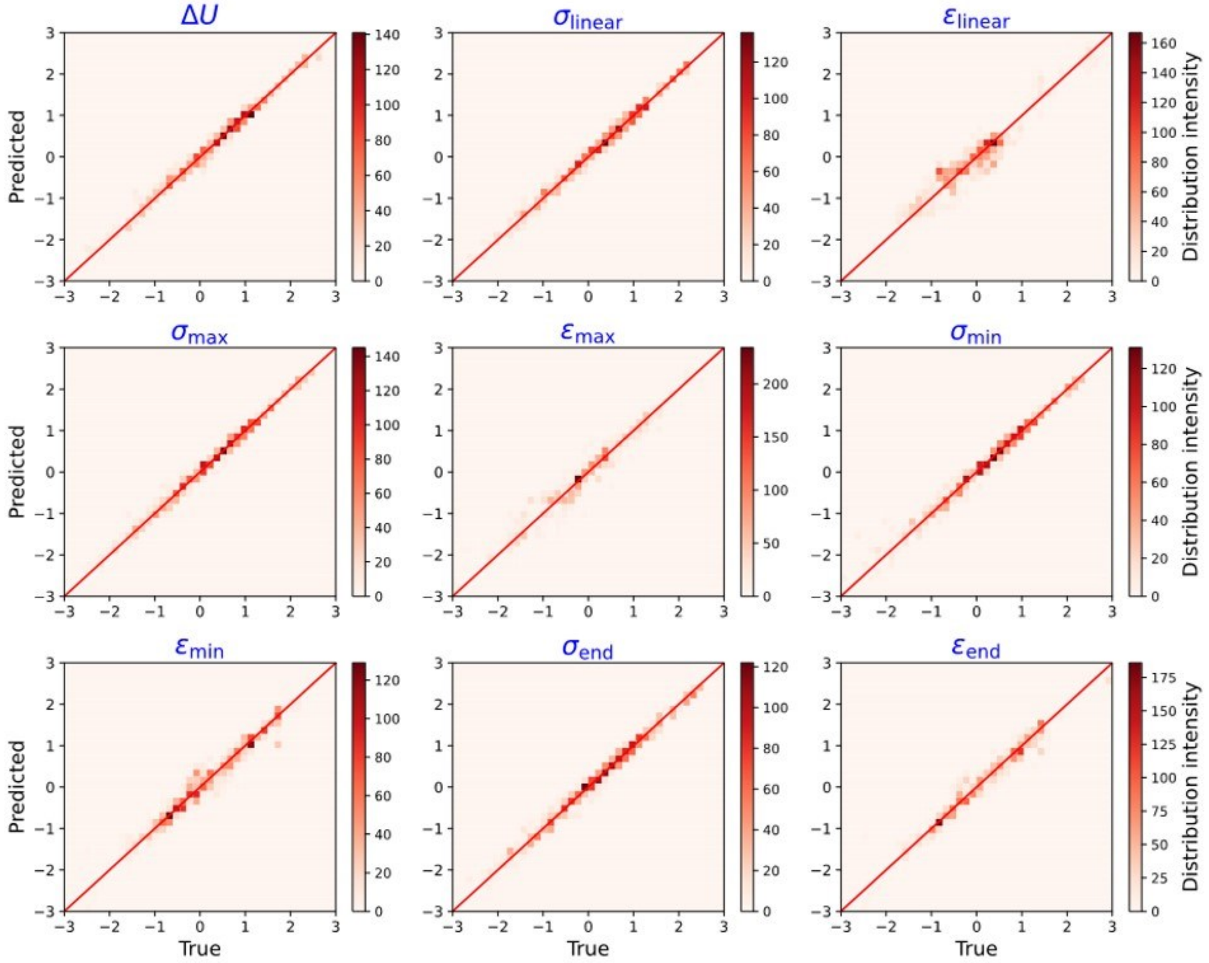

Supplementary Fig. 12 | Prediction accuracy of the forward validation module. Predicted versus true curve features offered by the forward surrogate model for the test set. Note that the output curve features are standardized.

Based on the predicted curve features, we can reconstruct the stress-strain curves associated with these features (Supplementary Note 1.1), to conduct a visual comparison between the predicted and true stress-strain curve. Supplementary Fig. 13 provides some examples of predicted versus true test curves offered by the forward validation module, where the predicted curves are reconstructed from their predicted curve features, and the predictions range over all types of stress-

train curves, including linear, plastic yielding response, buckling response, and multiple peak-and-valley response. It is notable that, for all the curves in the test set, the predicted and true curves largely overlap with each other, with a NRMSE that is smaller than 0.1. Once again, these results illustrate that the surrogate forward model can offer an accurate prediction of stress-strain curve for a given design of the lattice. This confirms that the forward model can be used as an accurate surrogate simulator and, hence, can be used to train the generative forward model (see Supplementary Note 7.3). Note that the ground-truth simulation engine itself cannot be used to train the generative model as it is not differentiable and, hence, does not enable back-propagation training—so that it is here necessary to replace the ground-truth simulator by a differentiable deep learning surrogate forward model.

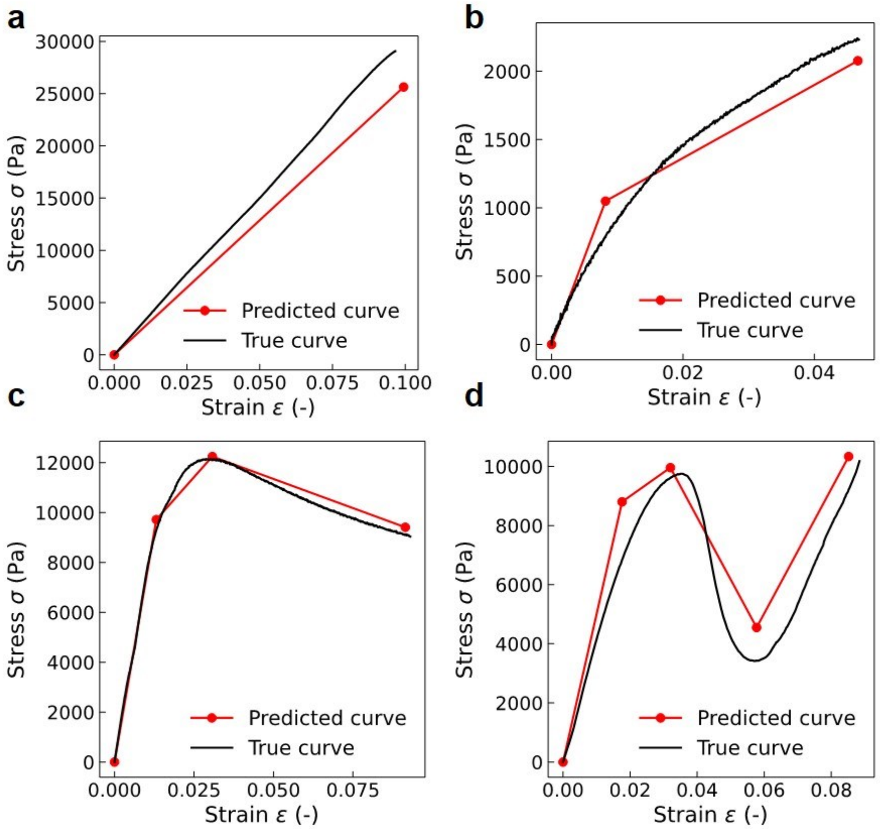

Supplementary Fig. 13 | Examples of predicted (red) versus true (black) test curves offered by the forward validation module. **a**, Linear response. **b**, Plastic yielding response. **c**, Buckling response. **d**, Multiple peak-and-valley response. The predicted curves are reconstructed from their predicted curve features.

Supplementary Note 7.3. Training of the inverse prediction module

Next, utilizing the pristine training dataset, we train the backward generative neural networks by connecting it to the surrogate forward model (see the model construction in Supplementary Note 1.1). In detail, by inputting the target stress-strain curve feature $\{\mathbf{X}^{\mathrm{T}}\}$, the backward generative model predicts a design candidate of the lattice $\{\mathbf{Y}\}$, which is then fed into the surrogate forward model to evaluate its corresponding curve feature $\{\mathbf{X}^{\mathrm{P}}\}$ for validation. As described in the previous

section, we defined the loss function as the mean squared error between $\{\mathbf{X}^{T}\}$ and $\{\mathbf{X}^{P}\}$ (f_c=($\mathbf{X}_{pred}$-$\mathbf{X}_{true}$)^2) and minimize the loss via gradient backpropagation, where the surrogate forward model is kept frozen (i.e., with fixed hyperparameters). Note that the dataset adopted in this training process is the pristine dataset (~1000 data points) without any data augmentation (Supplementary Note 6), where both the input and output the curve features $\{\mathbf{X}\}$ along with the information of curve type T_{curve}. The dataset was randomly split into training and test sets, where the test set contains 280 curves and accounts for 30% of the dataset. We conducted a 10-fold cross validation on the remaining training set to optimize the hyperparameters in the backward generative model. A SGD optimizer was adopted to minimize the loss function, with an initial learning rate of 0.01, a Nesterov momentum of 0.9, and a batch size of 32 curves. The learning rate was set to decay 1 order of magnitude after a patience of 10 training epochs. Supplementary Fig. 14a shows the test set loss as a function of the number of training epochs. After 200 training epochs, the test set loss converges to a miniscule level (~0.1).

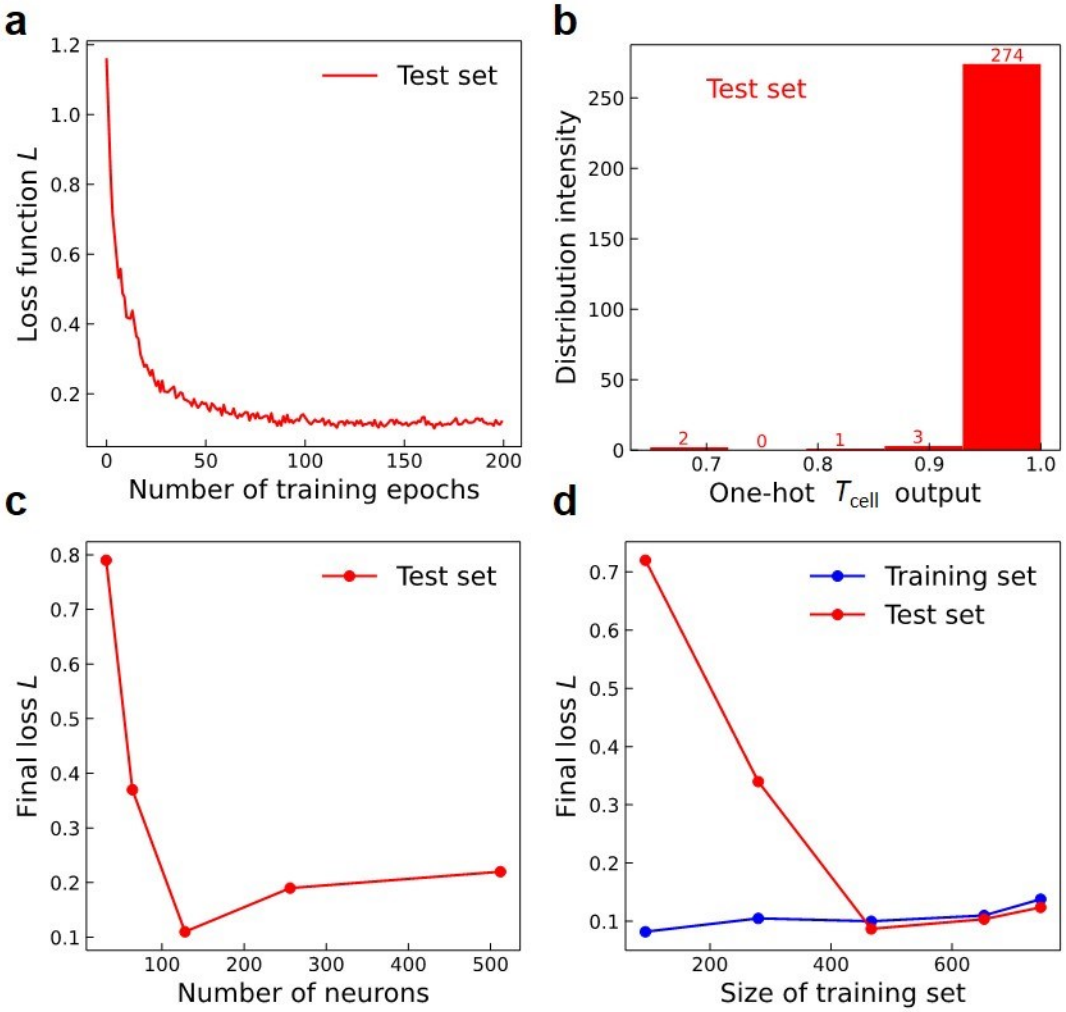

Supplementary Fig. 14 | Training results of the inverse prediction module. a, Inverse prediction loss L as a function of the number of training epochs for the test set. The test set contains 280 curves and accounts for 30% of the dataset. **b**, Distribution histogram of the one-hot-encoded T_{cell} output for the test set. Note that the backward generative model adopts a softmax activation for the T_{cell} output. **c**, Final test loss as L as a function of the number of neurons in the second hidden layer. Note that the number of neurons in the first hidden layer is set to be 2 times that of the second hidden layer. **d**, Final loss L (for both the training and test sets) as a function of the size of training set.

Note that, since the T_{cell} output of the generative model is based on a softmax activation function, the predicted T_{cell} values are not a standard one-hot representation (i.e., not an array composed of zero and one), which is likely to be outside the estimation range of the forward surrogate model. In order to make the predicted T_{cell} close to the standard one-hot representation, we designed a regularization term as the sum of $(1 - \max(T_{cell}))$ to restrict the T_{cell} output toward a one-hot representation. Supplementary Fig. 14b shows the distribution histogram of the one-hot-encoded T_{cell} output for the test set. As expected, the one-hot-encoded values of T_{cell} are mostly located in the range of [0.95, 1.0), with only 6 out of the 280 test curves being slightly smaller than 0.95. Thus, the predicted T_{cell} remains an approximation of a one-hot representation, and the small deviations of T_{cell} output are within the estimation range of forward surrogate model, given the fact that the forward model was trained by an augmented dataset with one-hot-encoded T_{cell} values ranging from 0.9 to 1 (Supplementary Note 7.2).

It is worth mentioning that we have optimized the inverse model performance by tuning all relevant training hyperparameters (to maximize the cross-validation accuracy). Supplementary Fig. 14c shows the final test loss as a function of the number of neurons in the second hidden layer, wherein the number of neurons in the first hidden layer is twice as large as in the second hidden layer, while other hyperparameters remain unchanged. We found that the test loss exhibits a minimum loss at 128 neurons, and that more neurons are not necessary to reduce the final test loss. Therefore, we selected herein 128 neurons in the second hidden layer to achieve an optimal balance between model accuracy and simplicity.

Finally, we investigated the influence of training size on the inverse model performance by constructing a learning curve. Supplementary Fig. 14d shows the final loss as a function of the size of training set for both the training and test sets. We selected five training sizes, i.e., 10%, 30%, 50%, 70%, 90% of the dataset, and randomly split the dataset into training and test sets. Due to the sample deficiency issue, the model initially exhibited a high test-loss at small training size. As the training size increases, the test loss decreased and finally reached a plateau when the training size exceeds 50% of the dataset, with a miniscule final loss of ~0.1 for both the training and test sets. This shows that the backward model does not exhibit any notable level of over- or under-fitting. We selected herein 70% of the dataset as training set to avoid the issue of sample deficiency that impairs the inverse model performance.

Once trained, the backward generative model aims to predict a lattice structure for a target stress-strain curve. To ensure the reliability of the inverse prediction module on prediction of architectures with tailored mechanical behaviors, we further investigated the prediction accuracy of the backward generative model. Supplementary Fig. 15 shows some representative stress-strain curve features of the structures generated by the backward model for the test set (predicted) as a function of the true stress-strain curve features used as target (where the test set contains 280 curves). Note the output curve features are standardized. For each feature, it is notable that the datapoints are largely located around the $y = x$ identity line. These results demonstrate that the backward generative model can indeed generate some structures that exhibit curve features that show a good match with the targeted input curve features. This confirms that the inverse prediction module is able to properly generalize, that is, it is able to generate structures exhibiting tailored

stress-strain curves—despite the fact that the model was never exposed to this stress-strain curve during its training.

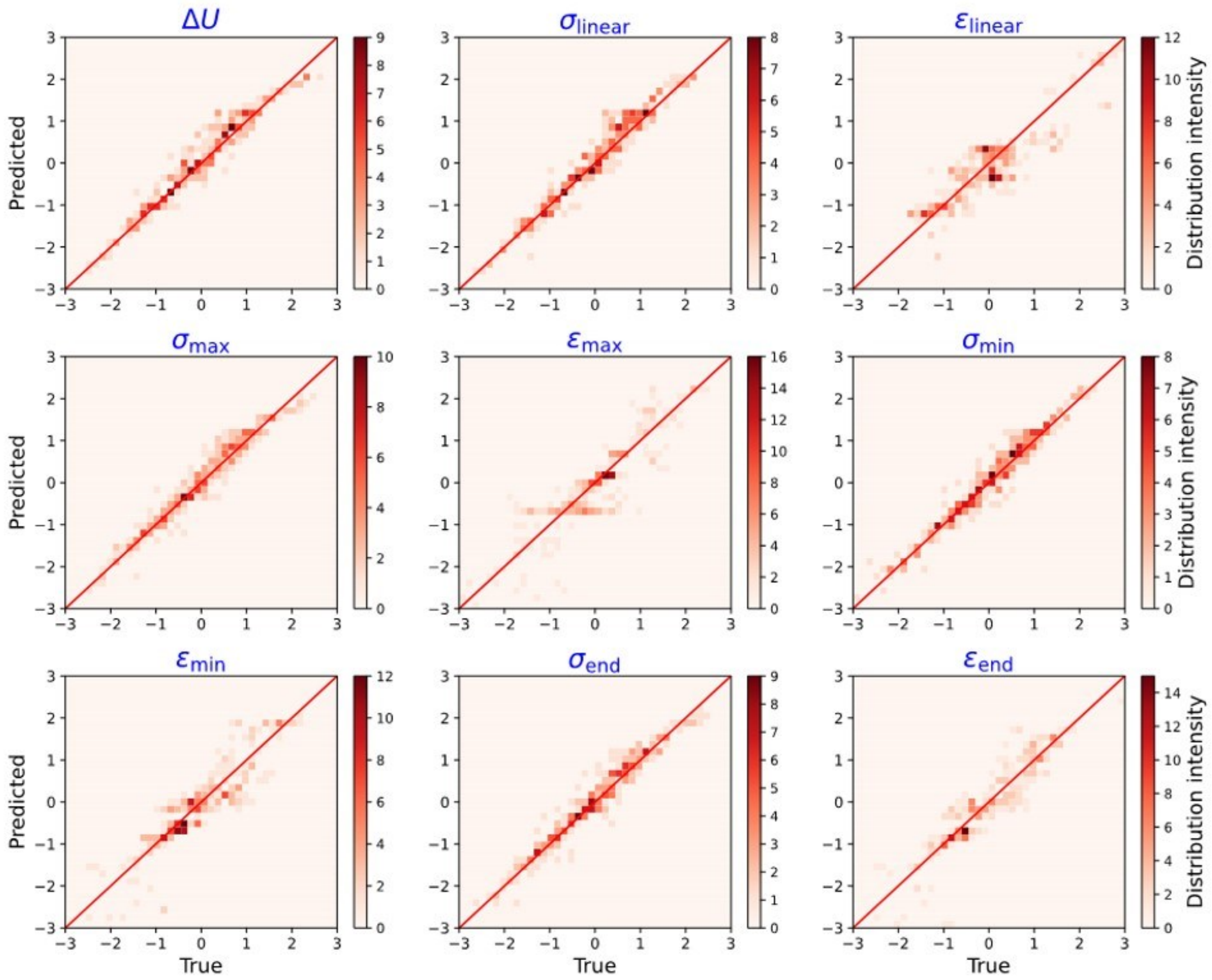

Supplementary Fig. 15 | Prediction accuracy of the inverse prediction module. Representative stress-strain curve features of the structures generated by the backward model for the test set (predicted) as a function of the true stress-strain curve features used as target. Note that the output curve features are standardized.

Based on the predicted curve features, we can reconstruct the stress-strain curves associated with these features (Supplementary Note 1.1), to conduct a visual comparison between the stress-strain curve of the generated structure and the target curve. Supplementary Fig. 16 provides some examples of stress-strain curves offered by the backward generative model, where the backward generative model generates a predicted structure that is fed into the forward surrogate model for validation, and the predicted curves are reconstructed from their predicted curve features offered by the forward surrogate model. The predictions range over all types of stress-train curves, including linear, plastic yielding response, buckling response, and multiple peak-and-valley response. It is notable that, for most curves in the test set, the generated and true curves largely overlap with each other, with a NRMSE smaller than 0.2. Again, these results illustrate that the backward generative model can accurately generate lattice design featuring tailored target stress-strain curves.

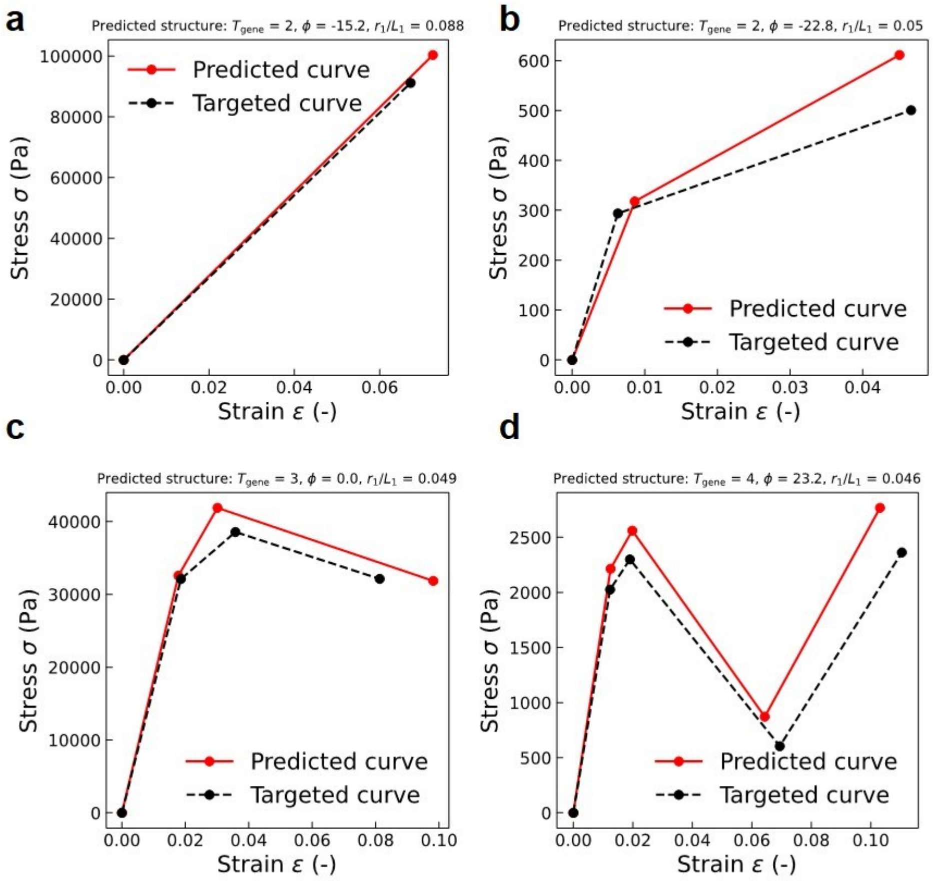

Supplementary Fig. 16 | Examples of predicted (red) versus true (black) test curves offered by the inverse prediction module. **a**, linear response. **b**, plastic yielding response. **c**, buckling response. **d**, multiple peak-and-valley response. By inputting a targeted curve, the inverse prediction module generates a predicted structure that is fed into the forward surrogate model for validation. The predicted curves are reconstructed from the predicted curve features offered by the forward surrogate model.

Supplementary Note 7.4. Training of multiple models under different initialization

In the previous sections, we described the training process of each individual neural network model, which is the foundation of optimal design selection. Here, we prepared five pairs of the forward surrogate neural network models and generative neural network models in our ML pipeline

(Supplementary Note 1.1). All the surrogate neural networks in the forward validation module and generative neural networks in the inverse prediction module are differentiated from other models in the same module with training hyperparameters, e.g., number of neurons, initialization, etc. Supplementary Fig. 17a shows a schematic of five generative training pipelines under different initialization, where each generative model is linked to its pairing surrogate models during the training and prediction process. The five individual surrogate forward models are obtained by different training initialization, which are kept frozen once trained and used to subsequently train their corresponding generative models, respectively.

In detail, we first conducted trainings of five surrogate neural network models with different initialization, while the augmented dataset is used. Supplementary Fig. 17b shows the test set loss as a function of the number of training epochs for five surrogate models, where the test set is randomly selected from the augmented dataset and contains 2808 curves (accounting for 30% of the augmented dataset). We found that the five trainings resulted in different final losses for the surrogate forward models, indicating they resulted in different surrogate neural network models. Nevertheless, all the losses eventually converge to a miniscule level between 0.05 and 0.1. We then used the five pretrained surrogate models to train their corresponding generative neural network models, respectively. Supplementary Fig. 17c shows the test set loss as a function of the number of training epochs for the generative models under the five trainings, where the test set is randomly selected from the pristine dataset and contains 280 curves (accounting for 30% of the pristine dataset). Similar to the training results of forward models, we found that each training resulted in a different generative model, with a distinguishable, convergent final loss between 0.1

and 0.2. Overall, relying on different training initialization, we obtain five different surrogate models and five different generative models.

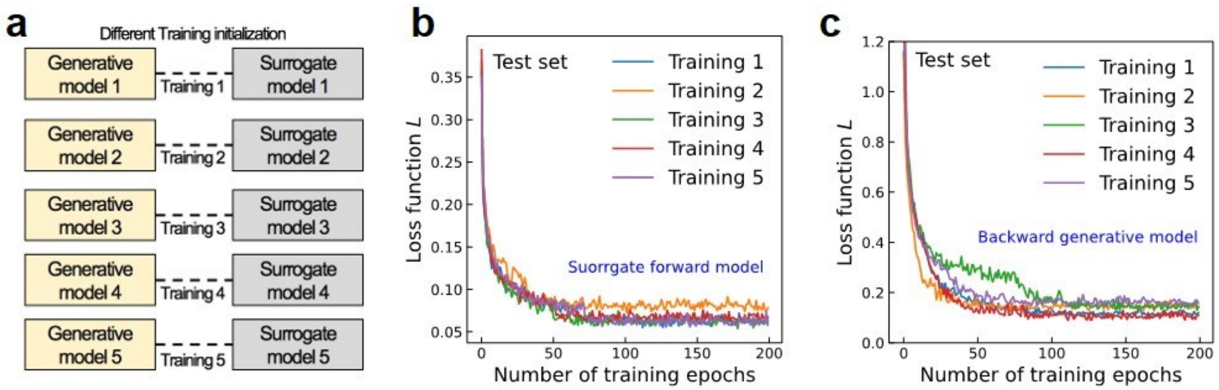

Supplementary Fig. 17 | Training results of multiple models with different initialization. a, Schematic illustrating five generative pipelines, wherein the five different surrogate forward models are obtained by different training initialization, which are used to train the five backward generative models, respectively. **b**, Test set loss as a function of the number of training epochs for the surrogate forward model under five different training initializations. The test set is randomly selected from the augmented dataset and contains 2808 curves, which accounts for 30% of the dataset. **c**, Test set loss as a function of the number of training epochs for the backward generative model under five different training initializations. The test set is randomly selected from the pristine dataset and contains 280 curves, which accounts for 30% of the dataset.

Supplementary Note 7.5. Evaluation of the multiple design candidates

Finally, we evaluate the optimal design from the five forward surrogate models and five backward generative models. To accomplish the evaluation task (Supplementary Fig. 1), we first input a

target curve feature $\{\mathbf{X}^{\mathrm{T}}\}$ to the five generative models. Each of the generative models predicts a design candidate of the lattice $\{\mathbf{Y}\}$, which is passed to the corresponding surrogate model to evaluate its predicted curve feature $\{\mathbf{X}^{\mathrm{P}}\}$. In detail, each surrogate model predicts five sets of curve feature given an input design candidate, which are averaged to obtain the final predicted curve feature for the candidate. In total, the ML pipeline output five design candidates and five corresponding averaged stress-strain curve feature for the selection of the optimal design. Supplementary Fig. 18 provides an example of the comparison between the input curve feature $\{\mathbf{X}^{\mathrm{T}}\}$ and five predicted curves and the averaged curve feature $\{\mathbf{X}^{\mathrm{P}}\}$ from one surrogate model. We noticed that the five sets of predicted curve feature of the generated structure exhibit a small deviation from each other (wherein the deviation offers an estimating of the uncertainty of the forward model prediction). These five estimated curves were then averaged to obtain an averaged curve feature $\{\mathbf{X}^{\mathrm{P}}\}$ estimation associated with the design candidate $\{\mathbf{Y}\}$, which is further used to calculate the NRMSE between the targeted curve $\{\mathbf{X}^{\mathrm{T}}\}$. Finally, the design candidate structure that features the minimum NRMSE between the averaged curve feature $\{\mathbf{X}^{\mathrm{P}}\}$ and the targeted curve feature $\{\mathbf{X}^{\mathrm{T}}\}$ is chosen as the optimal design. Overall, for our test target curves, we find that the optimal design generally exhibits an averaged estimated curve with both small NRMSE (< 0.1) and small uncertainty.

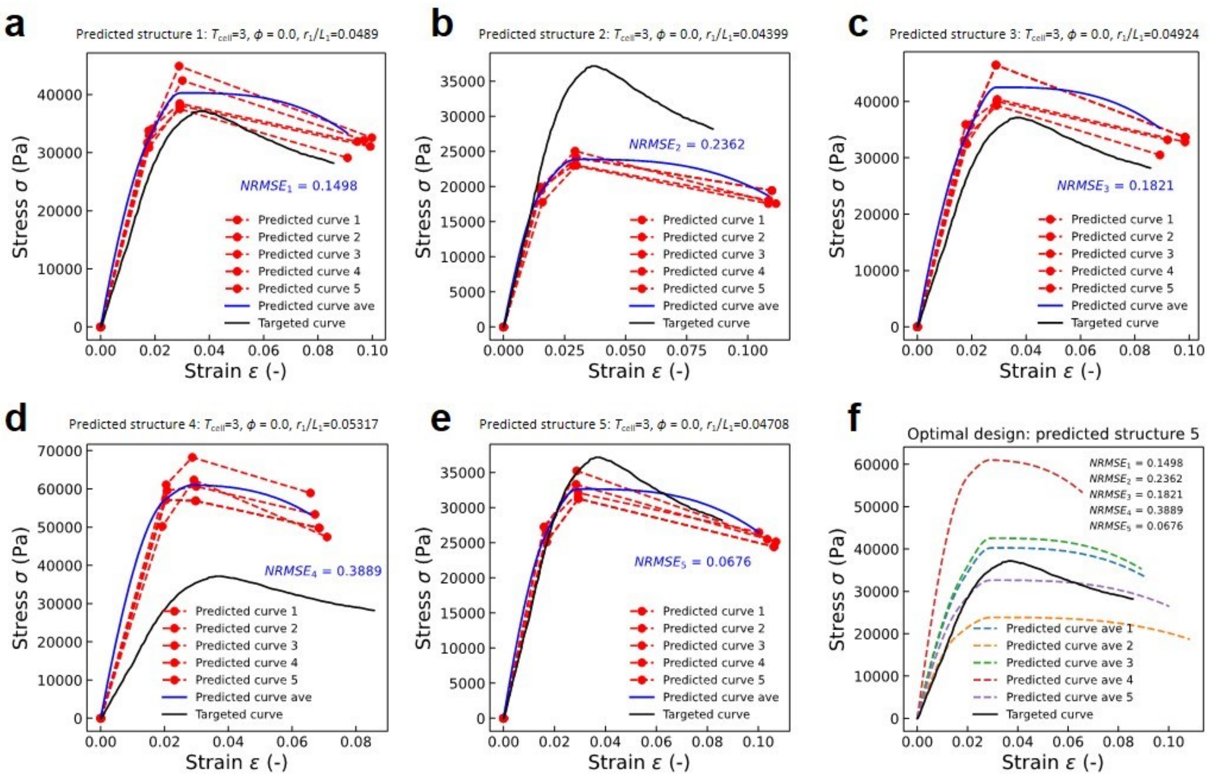

Supplementary Fig. 18 | Evaluation of multiple design candidates. a-e, Comparison between the target stress-strain curve with predicted stress-strain curve offered by the five pairs of generative-surrogate model, respectively. By inputting a targeted curve (black), each of the five generative model yields a predicted structure (indexed from 1 to 5, respectively), that is, a design candidate. The stress-strain curve of the generated structures is then reconstructed using curve feature estimated by all the five surrogate forward models, leading to five estimated curves per structure (red dash) in total. We then average the five sets of estimated curve feature (per structure) to obtain an averaged curve feature estimation (blue) associated with each of the generated structures. Finally, the normalized root-mean-square error (NRMSE) between the averaged estimated curve (blue) and the targeted curve (red) is calculated. **f**, Summary of the averaged estimated curve (dashed lines) associated with the five design candidates (i.e., the 5 generated structures) offered by the five generative models, respectively, wherein the targeted curve (solid)

is added for comparison. The design candidate featuring the lowest NRMSE (i.e., predicted structure 5 herein) is chosen as the optimal design.

Supplementary Note 7.6. Curve similarity test for the test curve

To test the machine learning design capability outside the seen domain (i.e., the domain of the training dataset), we performed recreation of the stress-strain curves not included in the training dataset. A curve shape similarity test between the test and training curves, by comparing these curves in terms of NRMSE, was conducted to demonstrate that the test curves is outside the domain of the training dataset. The results revealed that all test curves exhibit a widely spread distribution of NRMSE with considerable mean values between 0.5 and 0.62 (Supplementary Fig. 10d), verifying that every possible pair of the test and training curves is dissimilar. These findings confirm that the test curves are data not seen by the ML approach and support that the shown success is extendable to all stress-strain curve paths (Fig. 2b; example curve paths in Supplementary Fig. 4d) within the full design space defined in Fig. 2a.

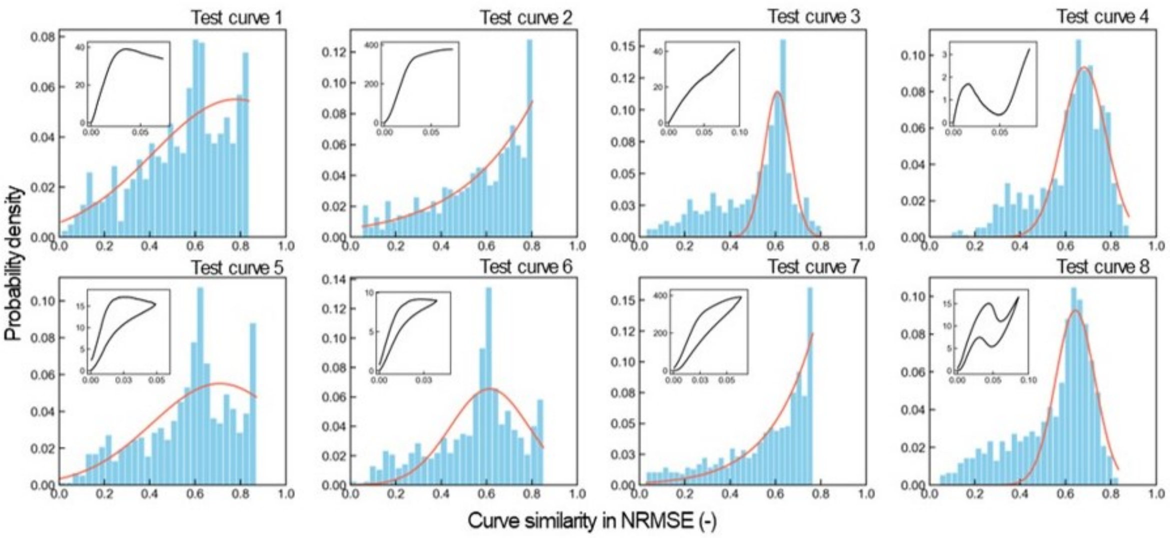

Supplementary Fig. 19 | Curve similarity test. Curve similarity test between the test and training curves in terms of NRMSE, where 0 imply identical curve pair with 1 implying completely dissimilar curve pair. Insets show the test curves (strain vs. stress in kPa). Red curves represent kernel density estimation.

Supplementary Note 8. Study of process variability

Supplementary Note 8.1. Effects of disparate process variabilities on ML prediction accuracy

Supplementary Note 8.1.1. Training dataset with stochastic noise and variability

We introduced noise to our training dataset to match variability in mechanical properties to that of other AM techniques. Herein, the variability was described by the variation in the elastic stiffness (i.e., $\eta = \frac{1}{n}\sum\frac{E_{\text{measured}}-E_{\text{fitted}}}{E_{\text{fitted}}}$, where E_{fitted} denotes a scaling relation between the elastic stiffness and the radius-to-length ratio of the inclined strut and n is the total number of data in our training dataset). Compared to the process variability of stereolithography printing approach used in this work (denoted by η_{SLA} = 13%), η of selective laser sintering (SLS) process is significantly higher (35%)[15-18]. Thus, we considered a range of η (25, 35, and 45%) to represent larger process variabilities than η_{SLA}.

These η were used to amplify variability in mechanical properties in our training dataset. This process started with generating a set of random numbers following a normal distribution with zero mean and standard deviation equal to a given η (Supplementary Fig. 20a). Then, a randomly selected value (x) from this set was used to amplify a distance between each curve feature and the corresponding fitted value in our training dataset (d_{ini}) so that a collection of increased distances (d_{new}) follows the normal distribution described by the given η (Supplementary Fig. 20b)—that is, $\frac{d_{\text{new}}}{d_{\text{ini}}} = \left(1 + \frac{x-\eta_{\text{SLA}}}{\eta_{\text{SLA}}}\right)$. This process was repeated for each η and led to three training datasets, of which each one represents different, larger process variability than that in our training dataset (η_{SLA}

= 13%). Supplementary Figs. 20c-d show representative probability distributions of before and after manipulation of the original training dataset using the process described above.

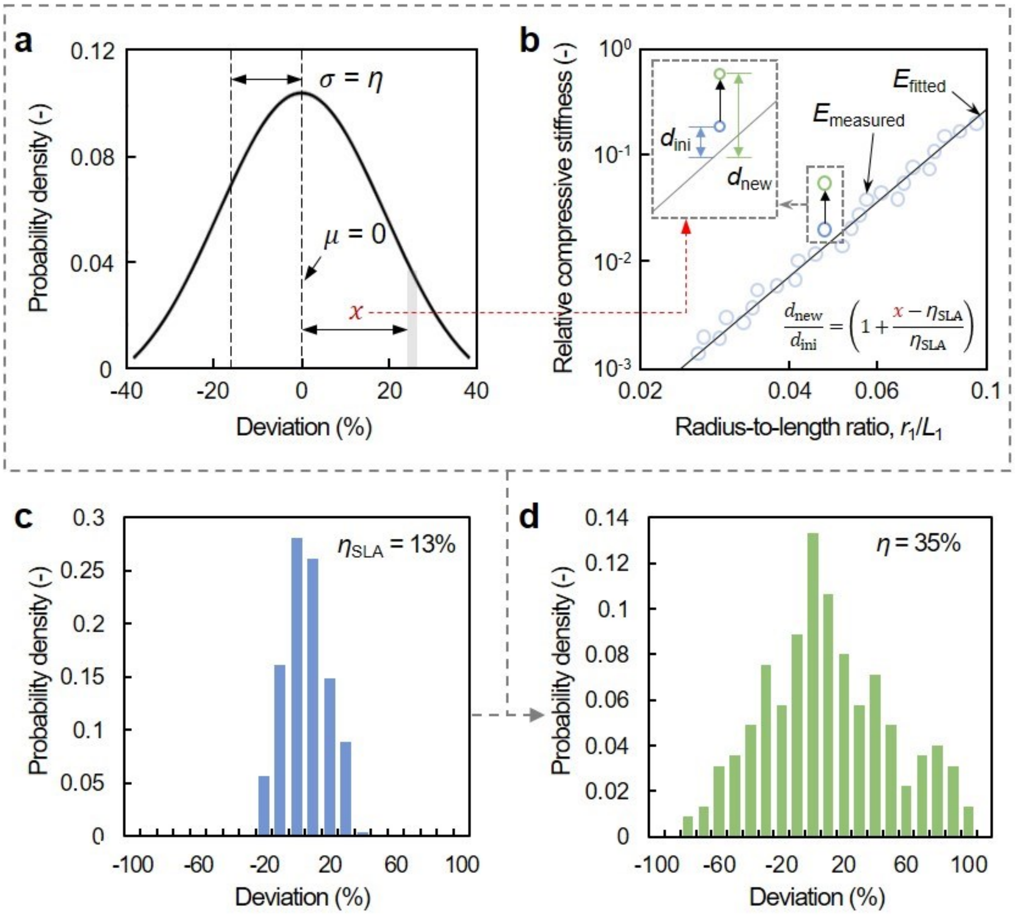

Supplementary Fig. 20 | Training datasets with stochastic noise and variability. a, A set of random values following a normal distribution with zero mean and standard deviation equal to a given η. **b**, Manipulation of curve features in our training dataset to match process variability described by a given η_P. A randomly selected value (x) from the set in a was used to amplify a distance between each curve feature and the corresponding fitted value (d_{ini}) so that increased distances (d_{new}) follow the normal distribution in **a**. **c**, Percentage deviation in the elastic stiffness in our training dataset, representing η_{SLA} of 13% (before manipulation). **d**, Percentage deviation in the elastic stiffness in a manipulated training dataset with η of 35% (after manipulation).

Supplementary Note 8.1.2. Evaluation of process variability: Prediction accuracy

Here the effects of disparate process variabilities on the overall prediction accuracy of our ML approach are presented. As done previously, we employed the 10-fold cross-validation technique to evaluate the overall prediction accuracy for each training dataset with larger process variabilities (η) as a function of data size.

The results show that the overall prediction accuracy scales with the data size and tends to converge to a plateau regardless of the level of process variability (Supplementary Fig. 21a). This implies that the model is capable of learning the underlying patterns even with the presence of considerable process variability. Furthermore, a degradation in the overall prediction accuracy was found to be marginal (~7%) when the process variability (η) increased from 13 to 35% (Supplementary Fig. 21b).

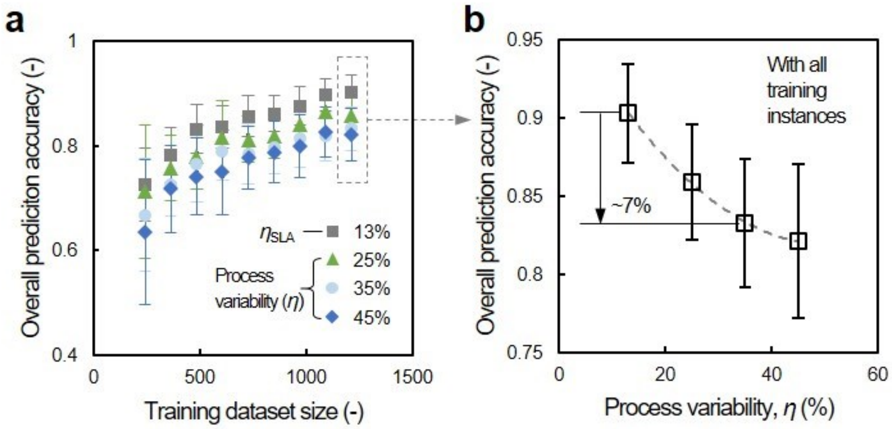

Supplementary Fig. 21 | Effects of the process variability on our ML approach. a, Overall prediction accuracy as a function of data size with various process variabilities (η) of 13, 25, 35, and 45 %. Monotonically increasing trends indicate applicability of our ML approach to disparate

process variability. **b**, Overall prediction accuracy as a function of process variability when all training instances are utilized. Error bars represent one standard deviation of uncertainty.

Supplementary Note 8.2. Case study: Using ML prediction in disrupted AM processes with larger variability

Supplementary Note 8.2.1. Gray-mask technique for realization of process variability

To experimentally mimic larger process variabilities, we integrated a gray-mask technique (Supplementary Fig. 22a) into the projection stereolithography system. The grayscale distribution for each digital mask was modulated to introduce non-uniformity in each layer and therefore realize larger geometric variations and defect levels. The gray-mask was formed by modulating the grayscale of each pixel with a random value in percentage. The range of these random percentages was determined by the target process variability. For instance, to replicate the property variability η of 35% (resembling SLS process), the range of the percentage is bounded between 20 and 90% (two representative gray masks shown in Supplementary Fig. 22b). Once the gray-masks were applied to all slices, the manipulated slices were utilized for printing.

The results from X-ray tomographic measurements on printed samples using the varied gray-mask technique are shown in Supplementary Fig. 22c. Both distributions of deviation in the strut diameter and the measured process variability in terms of the elastic stiffness agree well with those reported in prior studies[15-18] (the measured variability of ~32% vs. the reported variability of 35%) (Supplementary Figs. 22d-e).

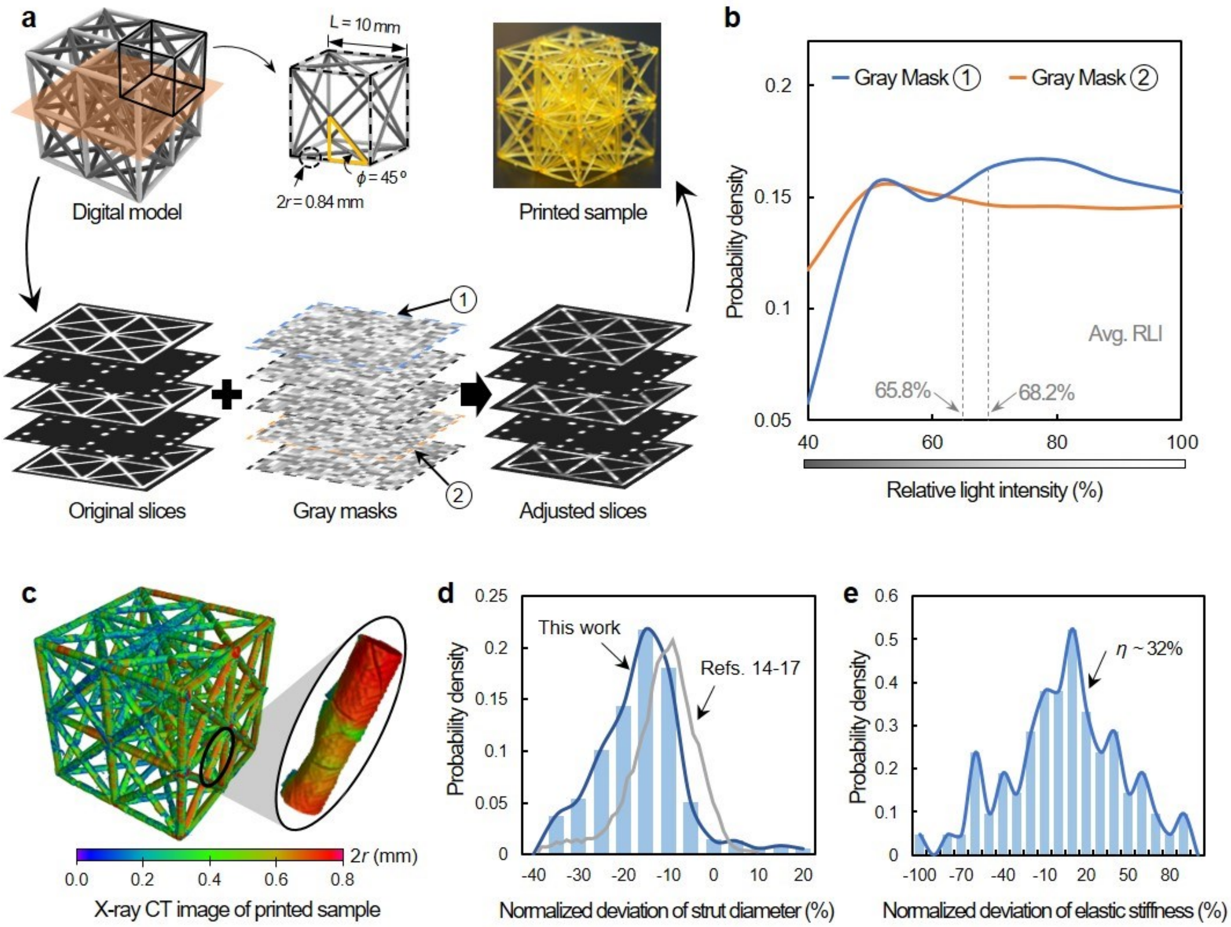

Supplementary Fig. 22 | Gray-mask technique and measurement of geometric and process variability in the printed sample. a, Schematic of gray-mask technique process allowing tuning of process variability. Intensity of white pixels in each slice from a digital model was stochastically varied to replicate geometric variation. **b**, Two representative gray masks applied to 2D sliced images shown in **a**. **c**, A representative X-ray image of the printed sample used to analyze a variation in strut diameter. A magnified view of a representative strut in the printed sample is shown to highlight the effects of process variability. **d**, Comparison of probability distributions of the normalized deviation of strut diameter between samples printed with the gray mask technique and data from the previous study[15-18]. **e**, Percentage deviation of the measured elastic moduli with

respect to the nominal value, confirming the measured process variability (η) of ~32 % in line with that found in literature[15-18].

Supplementary Note 8.2.2. Evaluation of process variability: Experimental testing results of ML designs

The effect of a representative larger process variability (η = 35%) on the curve recreating is examined here. A representative target stress-strain curve displaying linear-elastic response followed by elastic instability was fed into the ML pipeline. A total of ten samples representing the output design was fabricated using the developed gray-mask technique, and their cyclic compressive responses were measured and recorded as shown in Supplementary Fig. 23. The light-blue shaded region is an envelope covered by all tested stress-strain curves from the same printed design with the 35% process variability (i.e., η = 35%). For a comparison, the same target curve was inversely designed based on the process variability of the stereolithography process (η_{SLA} = 13%) used in this study (dark-blue colored uncertainty region in Supplementary Fig. 23). The results show that larger process variability introduces higher deviation from the tested stress strain curves and that our ML approach is sensitive to the process variability but still capable of re-creating the target curve with reasonable accuracy.

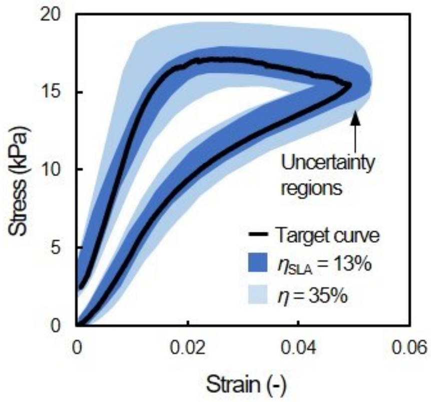

Supplementary Fig. 23 | Inversely designed, representative target stress-strain curve η = 35% (resembling the process variability of selective laser sintering process) and the gray-mask technique (light-blue shaded uncertainty region). For a comparison, the same target curve inversely designed based on the process variability of the stereolithography process (η_{SLA} = 13%) used in this study (dark-blue shaded uncertainty region).

Supplementary Note 8.3. Remedies for improvement in prediction accuracy

In summary, our analysis reveal that the proposed ML approach is indeed sensitive to process variability but still capable of reasonably learning the underlying response-to-design relationship even with considerable process variability. This was confirmed by a marginal degradation in prediction accuracy of our ML approach (Supplementary Fig. 13b) and the re-created stress-strain curves with a relatively small uncertainty (Supplementary Fig. 15). Thus, our ML approach is applicable to other AM platforms with larger process variability at a minimal cost of reliability. To ensure the reliability of our ML approach when substantial process variability is unavoidable, several remedies can be adopted as follows.

- Adding more training instances to a dataset – This strategy in general improves the prediction accuracy, as shown in Supplementary Fig. 21a. This is because ML becomes more robust by virtue of being trained on more examples[19] but this is not guaranteed as the quality of the newly added training instances may still contain spurious correlations degrading the robustness of ML.
- Data fusion, augmenting high-fidelity (e.g., experiment) data with low-fidelity (e.g., simulations) data[20,21] – The data fusion or augmentation allows combining the two data within the same training pipeline so that low- and high-fidelity data mutually augment, complement, and inform each other, thereby improving the prediction accuracy.
- Noise filtering or smoothing method[22] that eliminates irrelevant information (i.e., training instances with large deviation from a specified threshold) – This, commonly used in image and signal processing[19], has shown potential to improve the quality of the training dataset as well as prediction accuracy.

Supplementary Note 9. Inverse design of architected shoe midsole

Supplementary Note 9.1. Measurement of baseline curve

Stress-strain responses of a commercial shoe midsole (Manufacturer: Nike; product #: 908988 001) were measured to obtain baseline curves and used to inversely design the architected shoe midsole shown in Fig. 4d. We trimmed a sample from a heel section of the commercial shoe midsole (28×28×14 mm^3 in volume) (Supplementary Fig. 24a) and measured its stress-strain curves for strain rates $\dot{\varepsilon}$ from 10^{-3} to 0.4 s^{-1} representing from quasi-static to moderately dynamic regime (Supplementary Figs. 24b-d). We obtained the upper value of this strain rate range from a 75 kg man stepping on the sample at speed simulating real-life running scenario (i.e., $\dot{\varepsilon}$ = 0.302 s^{-1}). For all strain rates of interest, the mechanical responses of the sample were found to be rate independent, and we also found that samples trimmed from other sections of the commercial shoe midsole behave similar. Hence, we chose a stress-strain curve from the heel section tested at a strain rate of 0.3 s^{-1} (shown in Supplementary Fig. 24c) as a baseline curve and scaled this curve using the relative load distribution of the midsole during running[23] (shown in Fig. 4b) to obtain baseline curves for other sections.

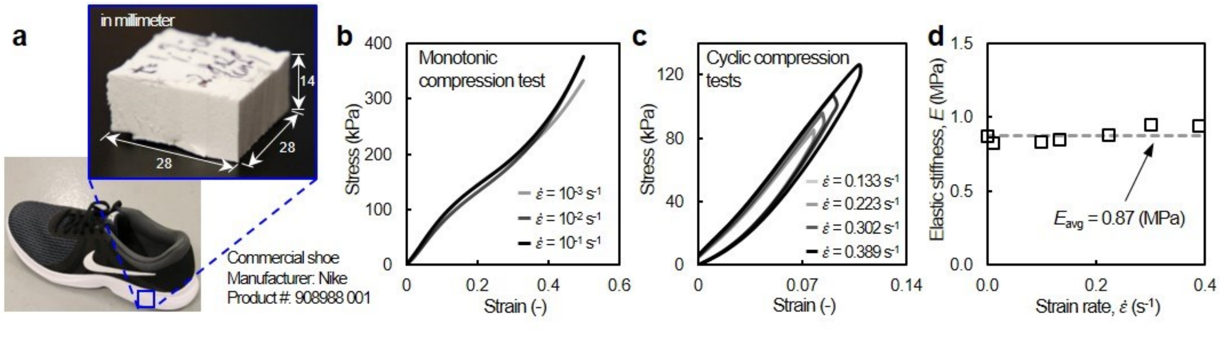

Supplementary Fig. 24 | Measurement of baseline curves from a commercial shoe midsole. a, Trimmed sample from the commercial shoe midsole. **b-d**, Measured responses of the trimmed sample for a range of strain rates.

Supplementary Note 9.2. Design rationale

We partitioned the architected shoe midsole into four sections: namely, toe, forefoot, midfoot, and heel (Fig. 4b). This is because each of these sections exhibits a different level of load during heel-toe running[23], and hence configuring them independently establishes a broad design potential to create the architected shoe midsole with spatially tailored mechanical responses for simultaneously satisfying multiple design targets. Herein, we focused on improving running performance through tailoring the baseline curves for specific targets. Inspired by the mechanism of augmented running[24], we started with designing a firmer heel section with an enhanced energy absorption by programming the elastic modulus by a factor of approximately 2.2 larger with 0.75 times less compressibility while maintaining a transmitted stress compared to the baseline response (Fig. 4d). This offers a rapid, enhanced energy absorption along with less compression, which in turn facilitates a fast weight transfer toward frontal sections of the midsole as well as improved propulsion. For the midfoot section, we replicated the midfoot response because of its negligible load magnitude compared to other sections (Fig. 4d). The forefoot section was tailored with a firmer and similarly dissipative behavior (~1.7 times stiffer and ~0.68 times less compressibility) compared to the baseline response (Fig. 4d). For the toe region, we tailored it by programming the stiffness by a factor of ~2.8 times larger with the same compressibility while exhibiting an improved propulsion and comfort (Fig. 4d).

Supplementary Note 9.3. Prediction for non-quasistatic strain rates

The mechanical properties of the base material (i.e., TMPTA), used for inverse design of the architected shoe midsole, exhibit the scaling relationships of the strain rate dependency, as shown in Supplementary Figs. 25a-b. While most of the stress-strain curves in this study are not loaded dynamically, the shoe midsole under various sporting events is loaded at different strain rate. The ML design of other loading rates can be achieved by transforming the stress-strain curve at other loading rates into the quasistatic case via the characterized scaling relationships of the base material. An as-printed sample based on the output design then will replicate the target stress-strain curve when tested at the desired strain rate.

To demonstrate this, a target curve for each section of the architected shoe midsole for running scenario (i.e., $\dot{\varepsilon}$ = 0.3s^{-1}) was transformed using the scaling relations of TMPTA described above (a representative adjusted stress-strain curve (i.e., toe section) shown in Supplementary Fig. 25c). The adjusted curves for each section were fed into our ML approach, and samples were created and printed based on the corresponding predicted design parameters. Supplementary Fig. 25d shows the measured curve of an inversely designed toe section at $\dot{\varepsilon}$ = 0.3s^{-1} compared to the target curve as an example, indicating satisfactory replicability (the measured curves for other sections in Fig. 4e). Closed-up views of each section of the as-printed sample inversely designed using this process and their design parameters are shown in Supplementary Fig. 25e. While other approach (e.g., training the ML program at all different loading rates) could also be implemented, the above strategy offers simple yet satisfactory results. In addition, results of a cyclic test at $\dot{\varepsilon}$ of 0. 3s^{-1} with

the number of cycles of 20 (i.e., N_{cycle} = 20) on the architected shoe midsole reveal that each tailored section exhibits marginal degradation over cycles (Supplementary Fig. 25f).

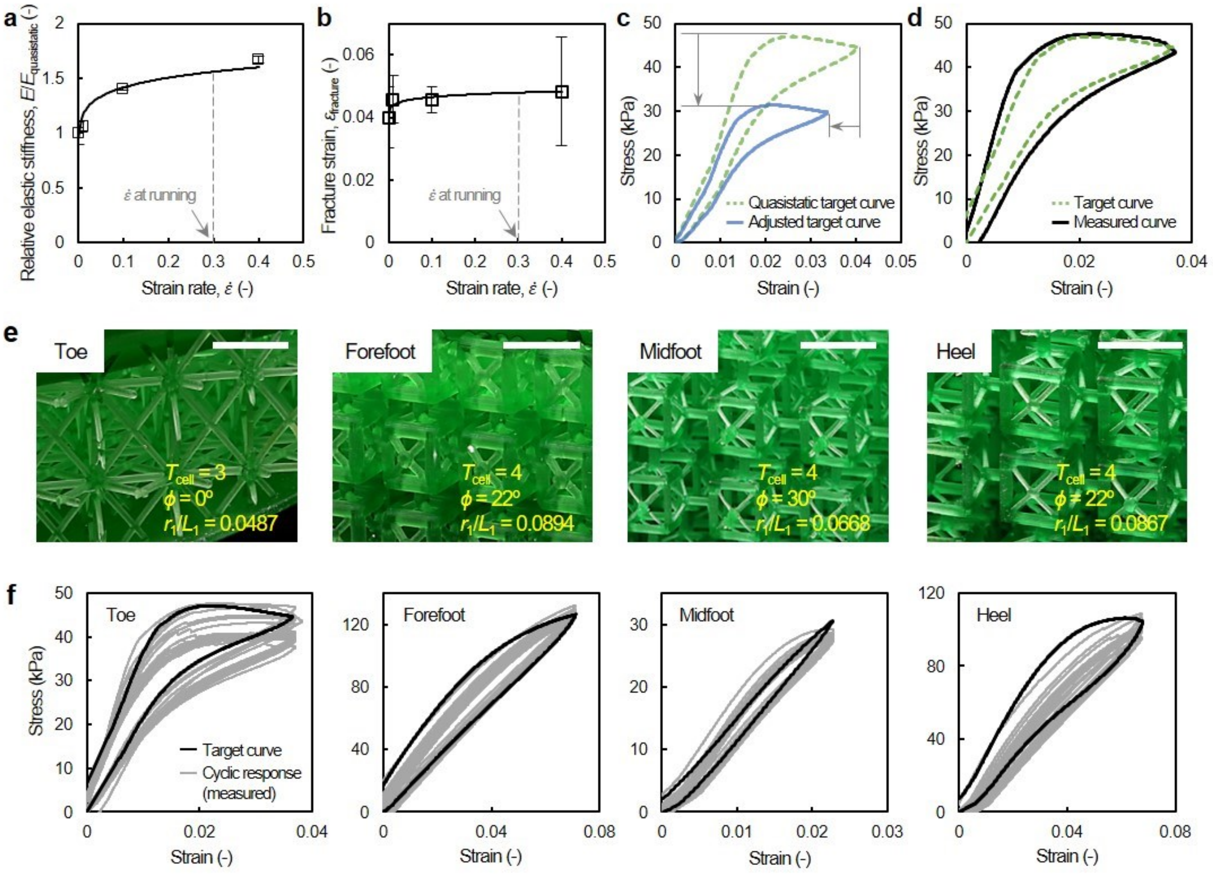

Supplementary Fig. 25 | Inverse design of the architected shoe midsole with strain rate effect. a-b, Rate dependency of bulk TMPTA on its material properties (i.e., $E/E_{quasistatic}$ versus $\dot{\varepsilon}$ and $\varepsilon_{fracture}$ versus $\dot{\varepsilon}$). Error bars represent one standard deviation of uncertainty. **c**, A representative target curve (i.e., toe section) adjusted for a higher strain rate (i.e., $\dot{\varepsilon}$ = 0.3s^{-1}) using scaling relations in rate dependency of TMPTA in **a** and **b**. **d**, Measured curve of an inversely designed toe section at $\dot{\varepsilon}$ = 0.3s^{-1} (black curve) compared to the (green dotted) original target curve. **e**, Spatially tailored sections of the inversely designed architected shoe midsole. The predicted design

parameters for each section are also listed. A scale bar is 10 mm. **f**, Measured cyclic response of the inversely designed midsole with many cycles (N_{cycle} = 20) at strain rate of 0.3s^{-1}.

Supplementary Note 10. Inverse design of compound lattices

Supplementary Note 10.1. Capability of compound lattices

Here we benchmarked three representative compound lattices generated with superposed unit cell topology-, strut radius-, cell size-gradients (G_1, G_2, G_4) against uniform lattices created by periodically repeating the cell types 1 and 3 as well as the octet-truss. A flexible base material (see Methods – Sample fabrication for detailed material formulation) was used to enable multiple layer collapses without failure under considerable deformation. As shown in Supplementary Fig. 26, our FE simulations revealed that the compound lattices were capable of bringing new features of stress-strain curves, including tunable tangent modulus, disparate peaks and valleys, and controllable hardening/softening effects, which is further beyond the boundary constrained by the uniform lattices. These uniform lattices exhibit a similar curve shape regardless of different topologies and varied geometric parameters.

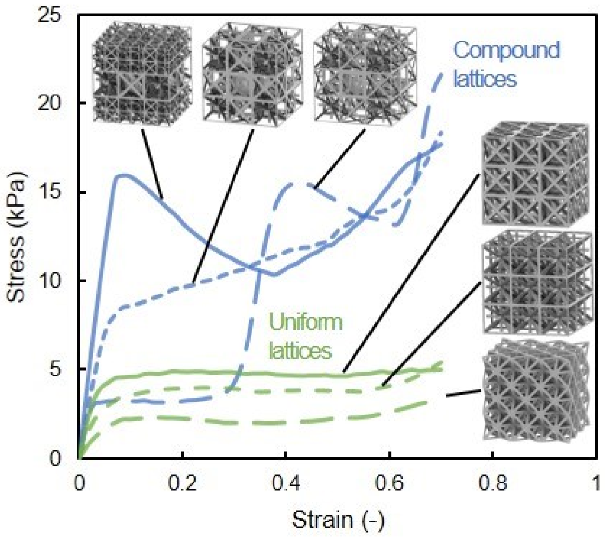

Supplementary Fig. 26 | Capability of the compound lattices. Comparison of stress-strain curve tailorability between the compound lattices created with superposed design gradients and the uniform lattices is shown.

Supplementary Note 10.2. Design gradients

The design gradients described in the main text characterize gradients in topological and geometric features of compound lattices: namely, the unit cell topology-, the strut radius ratio-, the inclined strut radius-, and the unit cell size-gradient (denoted by G_1, G_2, G_3, and G_4, respectively). To render as many superposed design gradients as possible while minimizing the size of a training dataset for our adjusted ML approach, we defined several gradient fields characterized by different spatial directional vectors (linear, concentrical, diagonal, and cylindrical). Supplementary Table 3 illustrates these predefined gradient fields in 3D heat maps, where red and blue colors represent the corresponding maximum and minimum, respectively. Applying dissimilar predefined gradient fields to the design gradients (i.e., G_1, G_2, G_3, and G_4) allows for realizations of a myriad of compound lattice designs to achieve advanced features of stress-strain curves.

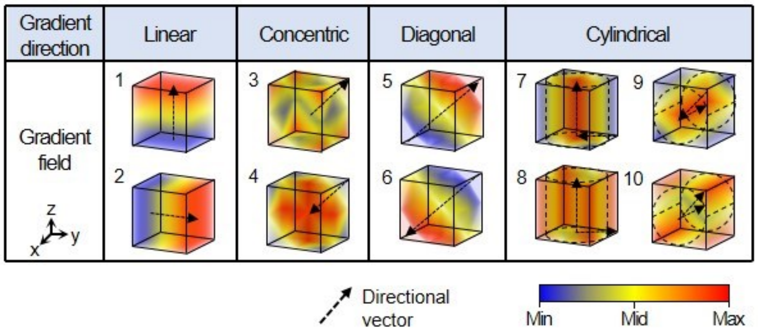

Supplementary Table 3 | Predefined gradient fields characterized by directional vectors. Arrow denotes the direction of the design gradient within the compound lattice.

Using the design gradients along with the predefined gradient fields described above, we set a baseline configuration to a 3×3×3 compound lattice. Each of G_1, G_2, and G_3 was therefore described by a 3×3×3 matrix representation, whereas a 1×3 vector representation was used to represent G_4. To use these mathematical descriptors of G_1, G_2, G_3, and G_4 as the output data of the ML approach, we compressed them into a 1×82 vector. Supplementary Fig. 27c and 27e illustrate a representative 3×3×3 compound lattice designed with superposed design gradients, where A, B, and C denote each layer.

Domains for the design gradients were constrained as follows. The unit cell topology gradient (G_1) was labeled by discrete values of 1, 3, and 5, representing the architectural cell types 1, 3, and 5, respectively. These cells were topological building blocks to construct a compound lattice. The strut radius ratio gradient (G_2), defined as the ratio of the inclined strut radius to the support strut radius (i.e., C or r_1/r_2), was represented by discrete values of 0.86, 1, 1.14, and 1.28, whereas the inclined strut radius gradient (G_3) was described by continuous values between 0.3 and 2.35 mm (corresponding to the relative density of each architectural cell from ~5 to ~40% with the unit cell length of 10 mm). The unit cell size gradient (G_4) was described by discrete values of $L_{\text{lattice}}/6$, $L_{\text{lattice}}/5$, $L_{\text{lattice}}/4$, and $L_{\text{lattice}}/3$, where L_{lattice} denotes the length of a compound lattice and is set to 30mm. The design gradient (G_4) was further simplified and classified by four sequence-independent stacking possibilities for a given volume as follows: (i) $L_{\text{lattice}}/3$–$L_{\text{lattice}}/6$–$L_{\text{lattice}}/6$–$L_{\text{lattice}}/3$ → 1; (ii) $L_{\text{lattice}}/6$–$L_{\text{lattice}}/6$–$L_{\text{lattice}}/3$–$L_{\text{lattice}}/6$–$L_{\text{lattice}}/6$ → 2; (iii) $L_{\text{lattice}}/4$–$L_{\text{lattice}}/4$–$L_{\text{lattice}}/6$–$L_{\text{lattice}}/3$ → 3; (iv) $L_{\text{lattice}}/6$–$L_{\text{lattice}}/4$–$L_{\text{lattice}}/6$–$L_{\text{lattice}}/4$–$L_{\text{lattice}}/6$ → 4. This representation can be

readily extended to a larger compound lattice in size by introducing a broader domain to each design gradient to express more complex gradients.

Supplementary Note 10.3. Training dataset for compound lattices

Approximately one hundred of compound lattices were created within the domain specified earlier for a training dataset generation. Their stress-strain curves in response to a quasi-static, monotonic compression were estimated by using FE simulations (see Methods — Finite element simulation). Each simulated curve was digitized into 46 curve features and used as the input data of the ML model (Supplementary Fig. 27a). For the output data, we compressed all matrices and vector representing the design gradients (G_1, G_2, G_3, and G_4) associated with a compound lattice into a 1×82 compressed vector (Supplementary Fig. 27e). For example, each of the topology-, the strut radius ratio-, the inclined strut radius-gradient (i.e., G_1, G_2, G_3), represented by the 3×3×3 matrix representation, was converted into a 1×27 vector. The unit cell size gradient (G_4) was described by the 1×3 vector representation denoting the four sequence-independent possibilities varying from 1 to 4. We then compressed all vectors and the classifier together to form a 1×82 vector.

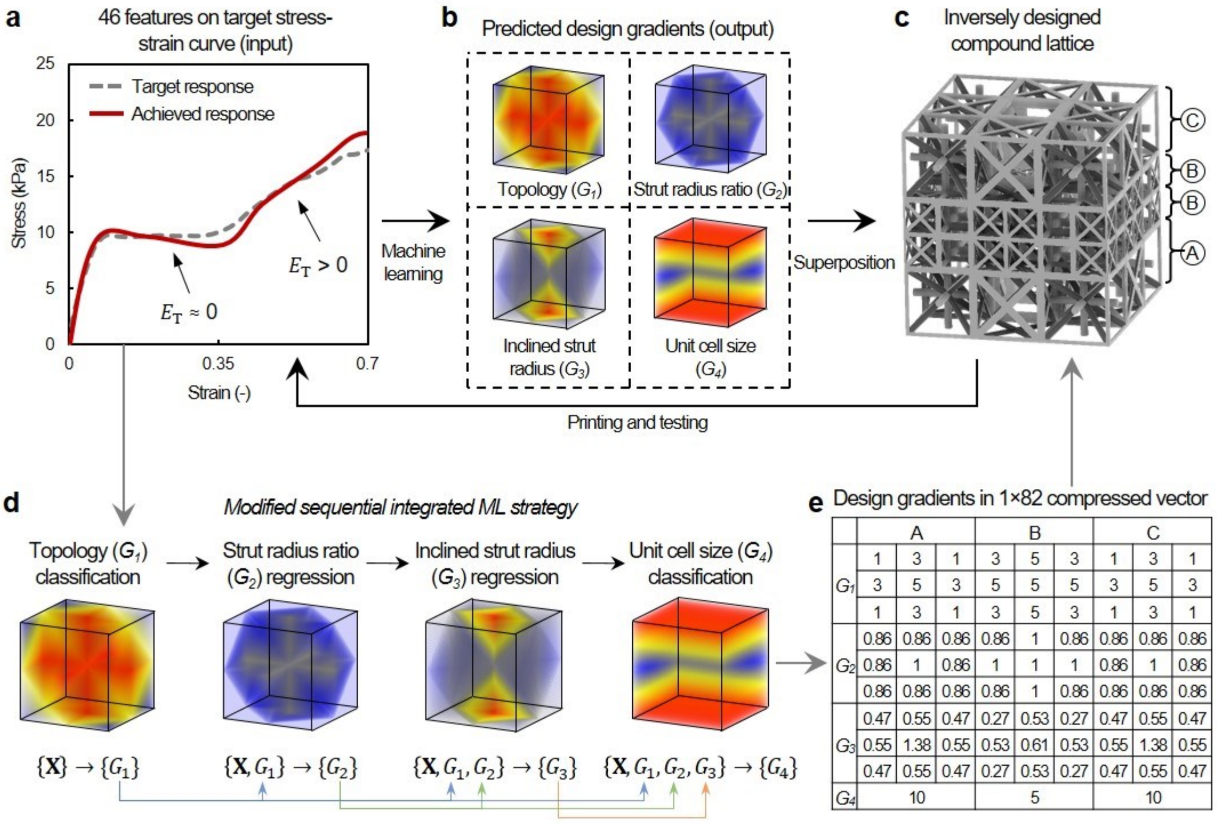

Supplementary Fig. 27 | Inverse design workflow of a compound lattice creation for enhanced stress-strain curve tailorability. a, Target stress-strain curve digitized with 46 feature points. **b**, Four design gradients: namely, the unit cell topology-, the strut radius ratio-, the inclined strut radius-, and the unit cell size-design gradients (G_1, G_2, G_3, G_4). **c**, Inversely designed compound lattice with superposed design gradients. **d**, Modified sequential integrated ML strategy for inverse design of compound lattices. **e**, The predicted design gradients represented in 1×82 compressed vector.

Supplementary Note 10.4. ML-predicted design gradients for target stress-strain curves in Figs. 5a-c

For the target stress-strain curves shown in Fig. 5a-c in the main text, the predicted design gradients are listed in Tables S6-S8, respectively. We adopted the coefficient of variance (COV), defined as the standard deviation divided by the mean of each design gradient, to quantify its spread (bar-type insets in Fig. 5a-c; the last column in Supplementary Tables 4-6). We used the predicted design gradients to visualize the corresponding 3D digital compound lattices shown in Fig. 5a-c.

Supplementary Table 4 | Design gradients for ML-designed compound lattices shown in Fig. 5a.

No.	Design gradient	Layer A			Layer B			Layer C			mean	std	COV
1	G_1	1	3	1	1	3	1	1	3	1	2.33	1.33	0.57
		3	5	3	3	5	3	3	5	3			
		1	3	1	1	3	1	1	3	1			
	G_2	1.14	1	1.14	1.14	1	1.14	1.14	1	1.14	1.05	0.09	0.09
		1	0.86	1	1	0.86	1	1	0.86	1			
		1.14	1	1.14	1.14	1	1.14	1.14	1	1.14			
	G_3	0.5	0.57	0.5	0.25	0.29	0.25	0.5	0.57	0.5	0.54	0.33	0.60
		0.57	1.59	0.57	0.29	0.8	0.29	0.57	1.59	0.57			
		0.5	0.57	0.5	0.25	0.29	0.25	0.5	0.57	0.5			
	G_4	5		5		10		5		5	6	2	0.33
2	G_1	1	3	1	1	3	1	1	3	1	2.93	1.49	0.51
		3	5	3	3	5	3	3	5	3			
		1	3	1	1	3	1	1	3	1			
	G_2	1.14	1	1.14	1.14	1	1.14	1.14	1	1.14	1.06	0.07	0.06
		1	1	1	1	1	1	1	1	1			
		1.14	1	1.14	1.14	1	1.14	1.14	1	1.14			
	G_3	0.57	0.57	0.57	0.57	0.57	0.57	0.57	0.57	0.57	0.63	0.17	0.27
		0.57	1.11	0.57	0.57	1.11	0.57	0.57	1.11	0.57			
		0.57	0.57	0.57	0.57	0.57	0.57	0.57	0.57	0.57			
	G_4		10			10			10		10	0	0
3	G_1	1	3	1	3	5	3	1	3	1	3	0	0

		3	5	3	5	5	5	3	5	3			
		1	3	1	3	5	3	1	3	1			
	G_2	0.86	0.86	0.86	0.86	1	0.86	0.86	0.86	0.86	0.90	0.60	0.07
		0.86	1	0.86	1	1	1	0.86	1	0.86			
		0.86	0.86	0.86	0.86	1	0.86	0.86	0.86	0.86			
	G_3	0.47	0.54	0.47	0.27	0.53	0.27	0.47	0.54	0.47	0.55	0.25	0.46
		0.54	1.38	0.54	0.53	0.612	0.53	0.54	1.38	0.54			
		0.47	0.54	0.47	0.27	0.53	0.27	0.47	0.54	0.47			
	G_4	10			5		5	10			7.5	2.5	0.33

1064

Supplementary Table 5 | Design gradients for ML-designed compound lattices shown in Fig. 5b.

No.	Design gradient	Layer A			Layer B			Layer C			mean	std	COV
4	G_1	1	3	5	1	3	5	1	3	5	3	1.63	0.54
		1	3	5	1	3	5	1	3	5			
		1	3	5	1	3	5	1	3	5			
	G_2	1.28	1.14	1	1.28	1.14	1	1.28	1.14	1	1.14	0.111	0.10
		1.28	1.14	1	1.28	1.14	1	1.28	1.14	1			
		1.28	1.14	1	1.28	1.14	1	1.28	1.14	1			
	G_3	0.55	0.5	1.4	0.55	0.5	1.4	0.55	0.5	1.4	0.82	0.41	0.51
		0.55	0.5	1.4	0.55	0.5	1.4	0.55	0.5	1.4			
		0.55	0.5	1.4	0.55	0.5	1.4	0.55	0.5	1.4			
	G_4	10			10			10			10	0	0
5	G_1	1	3	1	3	5	3	1	3	1	2.93	1.49	0.51
		3	5	3	5	5	5	3	5	3			
		1	3	1	3	5	3	1	3	1			
	G_2	1.14	1	1.14	1	1	1	1.14	1	1.14	1.04	0.06	0.06
		1	1	1	1	1	1	1	1	1			
		1.14	1	1.14	1	1	1	1.14	1	1.14			
	G_3	0.54	0.57	0.54	0.54	1.11	0.54	0.54	0.57	0.54	0.73	0.30	0.42
		0.57	1.38	0.57	1.11	1.38	1.11	0.57	1.38	0.57			
		0.54	0.57	0.54	0.54	1.11	0.54	0.54	0.57	0.54			
	G_4	10			10			10			10	0	0
6	G_1	3	3	3	3	3	3	3	3	3	3	0	0
		3	3	3	3	3	3	3	3	3			
		3	3	3	3	3	3	3	3	3			
	G_2	1.14	0.86	1.14	1.14	0.86	1.14	1.14	0.86	1.14	0.98	0.14	0.14
		0.86	0.86	0.86	0.86	0.86	0.86	0.86	0.86	0.86			
		1.14	0.86	1.14	1.14	0.86	1.14	1.14	0.86	1.14			
	G_3	0.4	0.48	0.4	0.4	0.48	0.4	0.4	0.48	0.4	0.53	0.25	0.48
		0.48	1.23	0.48	0.48	1.23	0.48	0.48	1.23	0.48			
		0.4	0.48	0.4	0.4	0.48	0.4	0.4	0.48	0.4			
	G_4	10			10			10			10	0	0

Supplementary Table 6 | Design gradients for ML-designed compound lattices shown in Fig. 5c.

No.	Design gradient	Layer A			Layer B			Layer C			mean	std	COV
7	G_1	1	1	1	1	3	1	1	1	1			
		1	3	1	3	5	3	1	3	1	1.59	1.06	0.67
		1	1	1	1	3	1	1	1	1			
	G_2	1	1	1	1	1	1	1	1	1			
		1	1	1	1	1.28	1	1	1	1	1.01	0.05	0.05
		1	1	1	1	1	1	1	1	1			
	G_3	0.47	0.47	0.47	0.47	0.47	0.47	0.47	0.47	0.47			
		0.47	1.23	0.47	0.47	1.43	0.47	0.47	1.23	0.47	0.56	0.26	0.46
		0.47	0.47	0.47	0.47	0.47	0.47	0.47	0.47	0.47			
	G_4	10			10			10			10	0	0
8	G_1	1	1	1	1	3	1	1	1	1			
		1	3	1	3	5	3	1	3	1	1.59	1.06	0.67
		1	1	1	1	3	1	1	1	1			
	G_2	0.86	0.86	0.86	0.86	0.86	0.86	0.86	0.86	0.86			
		0.86	0.86	0.86	0.86	1	0.86	0.86	0.86	0.86	0.87	0.03	0.03
		0.86	0.86	0.86	0.86	0.86	0.86	0.86	0.86	0.86			
	G_3	0.43	0.43	0.43	0.43	0.43	0.43	0.43	0.43	0.43			
		0.43	1.23	0.43	0.43	1.43	0.43	0.43	1.23	0.43	0.53	0.27	0.52
		0.43	0.43	0.43	0.43	0.43	0.43	0.43	0.43	0.43			
	G_4	10			10			10			10	0	0
9	G_1	1	1	1	1	3	1	1	1	1			
		1	3	1	3	5	3	1	3	1	1.59	1.06	0.67
		1	1	1	1	3	1	1	1	1			
	G_2	1.28	1.14	1.28	1.28	1.14	1.28	1.28	1.14	1.28			
		1.14	0.86	1.14	1.14	0.86	1.14	1.14	0.86	1.14	1.17	0.19	0.11
		1.28	1.14	1.28	1.28	1.14	1.28	1.28	1.14	1.28			
	G_3	0.40	0.60	0.40	0.40	0.60	0.40	0.40	0.60	0.40			
		0.60	1.23	0.60	0.60	1.43	0.60	0.60	1.23	0.60	0.59	0.27	0.46
		0.40	0.60	0.40	0.40	0.60	0.40	0.40	0.60	0.40			
	G_4	10			10			10			10	0	0

The ML-predicted design gradients for each target stress-strain curve in Fig. 5d-f are visualized using 3D heat maps in Supplementary Table 7 and listed in Supplementary Table 8. Supplementary Fig. 28 illustrates experimentally measured stress-strain curves of the inversely designed compound lattices, progressions of deformation at different strains of the as-fabricated samples, and their deformation fields estimated by FE simulations. Supplementary Movies 2-4 show a deformation process of these lattices synced with the corresponding stress-strain curves.

Supplementary Table 7 | 3D heat maps of design gradients for ML-designed compound lattices shown in Fig. 5d-f and their printed samples. 3×3×3 compound lattices, designed with superposition of the design gradients, were patterned to create 6×6×6 compound lattices.

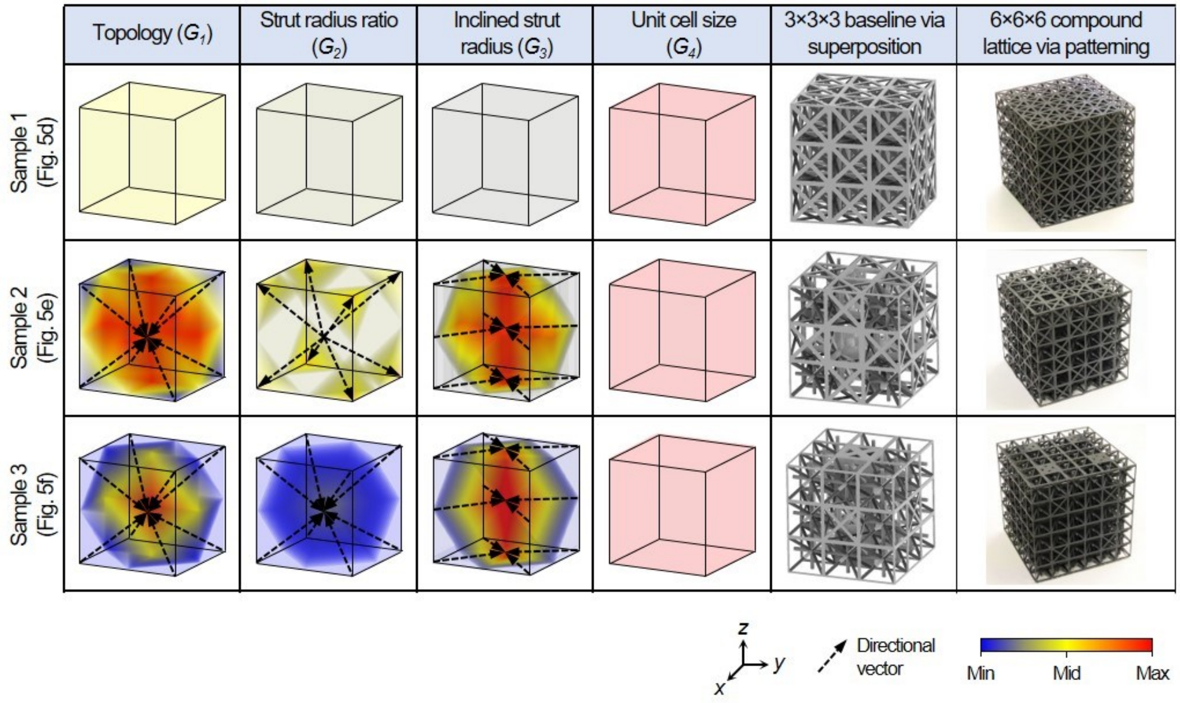

Supplementary Table 8 | Design gradients for ML-designed compound lattices shown in Fig. 5d-f.

Sample No.	Design gradient	Layer A			Layer B			Layer C			Mean	Std	COV
Sample 1 (Fig. 5d)	G_1	3	3	3	3	3	3	3	3	3	3	0	0
		3	3	3	3	3	3	3	3	3			
		3	3	3	3	3	3	3	3	3			
	G_2	1	1	1	1	1	1	1	1	1	1	0	0
		1	1	1	1	1	1	1	1	1			
		1	1	1	1	1	1	1	1	1			
	G_3	0.57	0.57	0.57	0.57	0.57	0.57	0.57	0.57	0.57	0.57	0	0
		0.57	0.57	0.57	0.57	0.57	0.57	0.57	0.57	0.57			
		0.57	0.57	0.57	0.57	0.57	0.57	0.57	0.57	0.57			
	G_4	10			10			10			10	0	0
Sample 2 (Fig. 5e)	G_1	1	3	1	3	5	3	1	3	1	2.926	1.489	0.509
		3	5	3	5	5	5	3	5	3			
		1	3	1	3	5	3	1	3	1			
	G_2	1.14	1	1.14	1	1	1	1.14	1	1.14	1.041	0.064	0.061
		1	1	1	1	1	1	1	1	1			
		1.14	1	1.14	1	1	1	1.14	1	1.14			
	G_3	0.54	0.57	0.54	0.54	1.11	0.54	0.54	0.57	0.54	0.726	0.303	0.416
		0.57	1.38	0.57	1.11	1.38	1.11	0.57	1.38	0.57			
		0.54	0.57	0.54	0.54	1.11	0.54	0.54	0.57	0.54			
	G_4	10			10			10			10	0	0
Sample 3 (Fig. 5f)	G_1	1	1	1	1	3	1	1	1	1	1.593	1.063	0.668
		1	3	1	3	5	3	1	3	1			
		1	1	1	1	3	1	1	1	1			
	G_2	0.86	0.86	0.86	0.86	0.86	0.86	0.86	0.86	0.86	0.865	0.026	0.031
		0.86	0.86	0.86	0.86	1	0.86	0.86	0.86	0.86			
		0.86	0.86	0.86	0.86	0.86	0.86	0.86	0.86	0.86			
	G_3	0.43	0.43	0.43	0.43	0.43	0.43	0.43	0.43	0.43	0.526	0.274	0.521
		0.43	1.23	0.43	0.43	1.43	0.43	0.43	1.23	0.43			
		0.43	0.43	0.43	0.43	0.43	0.43	0.43	0.43	0.43			
	G_4	10			10			10			10	0	0

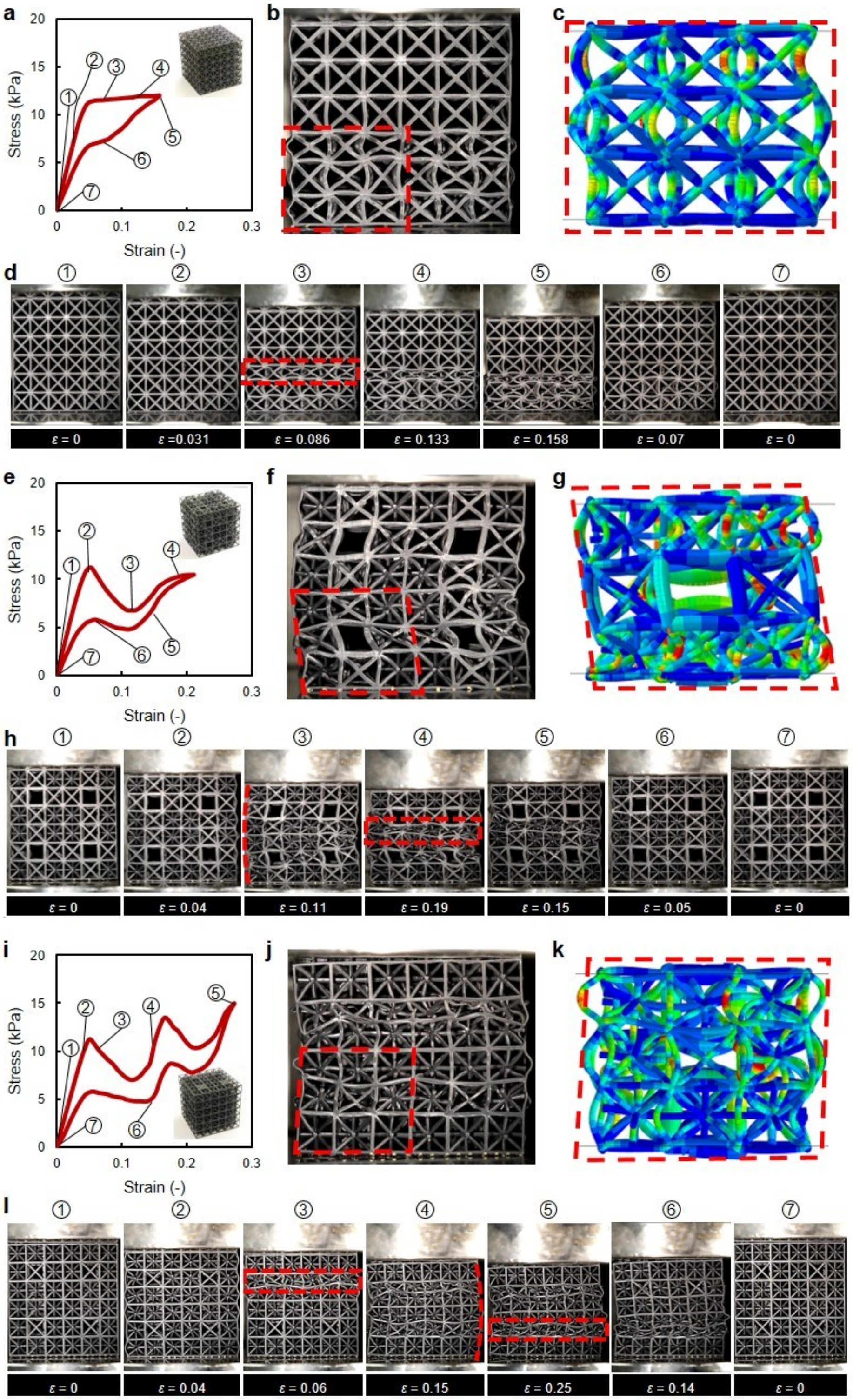

Supplementary Fig. 28 | Experimentally measured stress-strain curves of ML-designed compound lattices shown in Fig. 5d-f and progression of deformation at different strains of

the printed samples. a-d, Sample 1 shown in Fig. 5d. **e-h**, Sample 2 shown in Fig. 5e. **i-l**, Sample 3 shown in Fig. 5f. FE simulations for each lattice were performed and showed good agreements in deformation observed in experiment.

Supplementary Note 10.5. Details of energy absorption vs. strength property map

Supplementary Table 9 provides values for the normalized energy absorption vs. transmitted strength property map (that is, $(U/E_s)/\bar{\rho}$ vs. $(\sigma_{cr}/\sigma_{ys})/\bar{\rho}$) presented in Supplementary Fig. 29. To compare ML-designed compound lattices with the previously reported energy absorbing materials[25-31], we normalized the volumetric energy absorption ($U = \int \sigma d\varepsilon$) by the Young's modulus of the corresponding parent material (E_s) and the relative density ($\bar{\rho}$) and normalized the maximum transmitted strength (σ_{tr}) by yield strength of the corresponding parent material (σ_{ys}) and the relative density ($\bar{\rho}$). All data used was from quasi-static stress-strain responses with strain ranging from 0 to 0.7. These normalization yields a fair comparison of materials considered independent of parent material properties as well as a density of these materials.

In addition, an envelope covering the training dataset used to inversely design compound lattices associated with superposed design gradients, would be expanded if all possible configurations of the compound lattices were mapped out based on the tunable range of the design gradients (G_1, G_2, G_3, G_4), and this indicates a potential of a broader tunability of stress-strain curves.

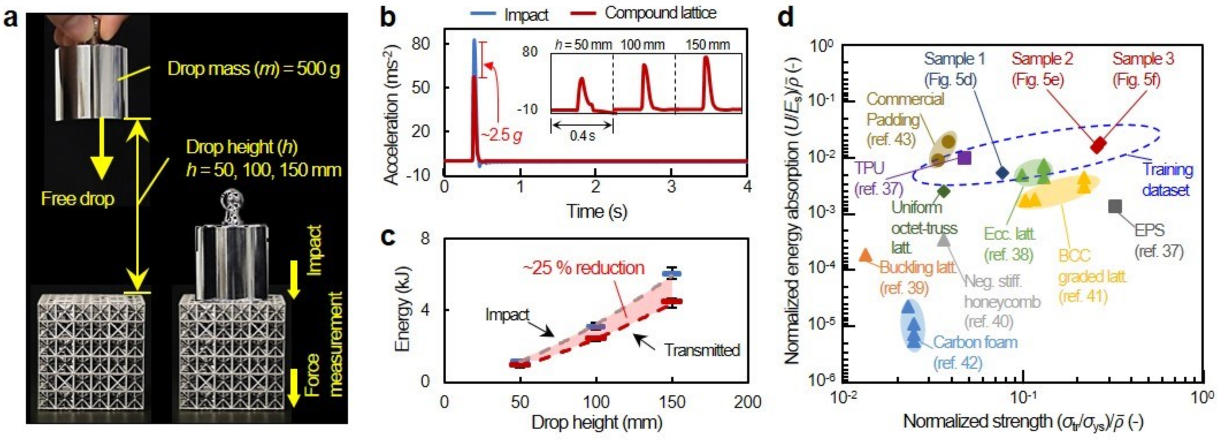

Supplementary Fig. 29 | Energy absorption characteristics of the ML-designed compound lattice. a, Drop test setup with different heights on the as-fabricated compound lattice shown in **Fig. 5f**. **b**, Acceleration-vs-time curve of the compound lattice with the dead weight dropped from $h = 150$ mm, revealing a reduced peak acceleration (~2.5g). An inset displays acceleration-vs-time curves of the compound lattice with different drop heights of h = 50, 100, 150 mm. **c**, Potential energy recorded from the force transducer for the compound lattice with different drop heights. The error bars indicate standard deviations from multiple ($N > 3$) measurements. A significant gap (shaded area) indicates energy absorption capability of the ML-designed compound lattice as a potential padding material. **d**, A normalized energy absorption-transmitted strength pair (i.e., $(U/E_s)/\bar{\rho}$ vs. $(\sigma_{tr}/\sigma_{ys})/\bar{\rho}$) of the ML-designed compound lattices compared with the state-of-the-art energy absorbing materials[37-43] as well as uniform lattices.

Supplementary Table 9 | The normalized energy absorption – strength pairs (i.e., $(U/E_s)/\bar{\rho}$ versus $(\sigma_{tr}/\sigma_{ys})/\bar{\rho}$) of the ML-designed compound lattices against the previously reported energy absorbing materials.

Material	Effective properties					Bulk material properties		Ref.
	$\bar{\rho}$ (-)	σ_{cr} (kPa)	$U_{loading}$ (kJ m^{-3})	$(\sigma_{tr}/\sigma_{ys})/\bar{\rho}$ (-)	$(U/E_s)/\bar{\rho}$ (-)	σ_{ys} (MPa)	E_s (MPa)	
TPU Twin Hemispheres Skydex	0.079	210	80	4.75×10^{-2}	1.01×10^{-2}	56	100	Ref[25]
EPS foam	0.062	1400	520	3.23×10^{-1}	1.40×10^{-3}	70	6000	
Eccentric lattice	0.1	1000	1000	1.31×10^{-1}	7.41×10^{-3}	76.5	1350	Ref[26]
	0.1	1000	900	1.31×10^{-1}	6.67×10^{-3}			
	0.1	1000	600	1.31×10^{-1}	4.44×10^{-3}			
	0.1	750	675	9.80×10^{-2}	5.00×10^{-3}			
Buckling lattice	0.09	131.18	39.24	1.34×10^{-2}	1.90×10^{-4}	108.8	2300	Ref[27]
Negative stiffness honeycomb	0.177	321.97	99.29	3.65×10^{-2}	3.55×10^{-4}	50	1582	Ref[28]
BCC graded lattice	0.19	945	549.5	1.04×10^{-1}	1.75×10^{-3}	48	1650	Ref[29]
	0.19	2000	940	2.19×10^{-1}	3.00×10^{-3}			
	0.19	1053	562	1.15×10^{-1}	1.79×10^{-3}			
	0.19	2000	1371	2.19×10^{-1}	4.37×10^{-3}			
Carbon foam	0.004	7	2.45	2.30×10^{-2}	2.22×10^{-5}	76	27600	Ref[30]
	0.004	7.5	1.23	2.47×10^{-2}	1.11×10^{-5}			
	0.004	7.5	0.8167	2.47×10^{-2}	7.40×10^{-6}			
	0.004	7.5	0.6125	2.47×10^{-2}	5.55×10^{-6}			
Hard foam (Riddell)	0.079	170	150	3.84×10^{-2}	1.90×10^{-2}	56	100	Ref[31]
Soft foam (Riddell)	0.079	150	72.5	3.39×10^{-2}	9.18×10^{-3}			
Sample 1 (Fig. 5d)	0.1	4.74	3.21	7.66×10^{-2}	5.40×10^{-3}	0.619	5.95	This work
Sample 2 (Fig. 5e)	0.1	15.94	9.00	2.58×10^{-1}	1.51×10^{-2}			
Sample 3 (Fig. 5f)	0.1	16.68	10.94	2.69×10^{-1}	1.84×10^{-2}			
Uniform octet-truss lattice	0.1	2.25	1.51	3.64×10^{-2}	2.54×10^{-3}			

Supplementary Note 11. Details of FE simulations

Supplementary Note 11.1. Choice of element type

Architected materials made of our cells with the relative density ($\overline{\rho}$) higher than 25% will converge to those of a solid (see CAD models in Supplementary Fig. 30a), as do their mechanical behavior. A coverage from such materials accounts for ~8.5% of the entire area covered by our cells for $0 \leq \overline{\rho} \leq 0.4$ (Supplementary Fig. 6b). Therefore, the majority of training data and inverse design is considered within $\overline{\rho} \leq 25\%$ and is responsible for ~91.5% of the entire area.

Supplementary Figs. 30b-c shows a comparison of simulated mechanical properties between solid and Timoshenko beam model for $\overline{\rho} \leq 25\%$. For $\overline{\rho} \leq 15\%$, Timoshenko beam represents the solid model reasonably well (~90% accuracy), whereas it underestimates by ~20% for $\overline{\rho} \geq 25\%$. This information is used in our training dataset where at $\overline{\rho}$ higher than 25%, solid model is used in place of Timoshenko beam.

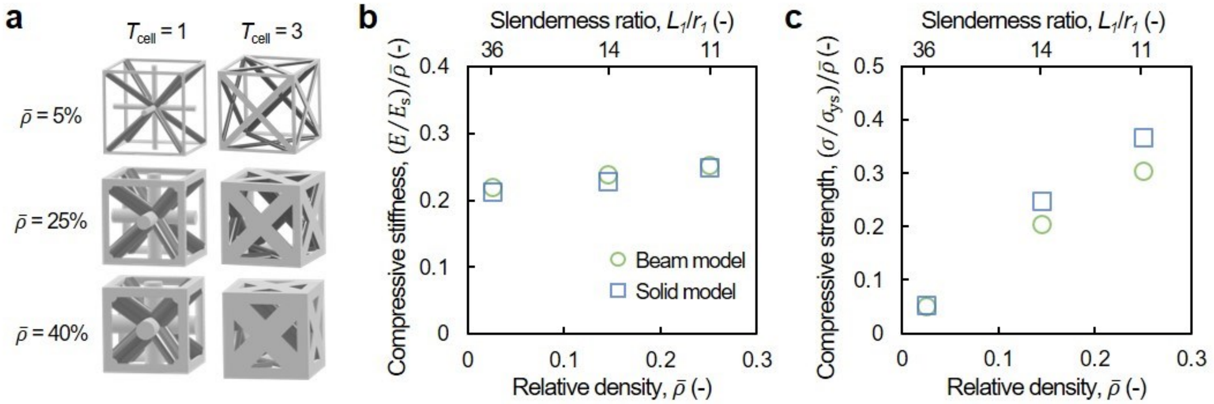

Supplementary Fig. 30 | Comparison of simulated mechanical properties between Timoshenko beam and solid models. a, Representative CAD models made with different relative densities ($\bar{\rho}$). **b-c**, Comparison of simulated compressive mechanical properties between Timoshenko beam and solid model for $\bar{\rho} \leq 0.25$.

Supplementary Note 11.2. Constituent material models

The constituent material models in FE simulations described in Methods reasonably simulates measured stress-strain curves of dogbone-shaped samples made of each base material, as shown in Supplementary Fig. 31. Mechanical properties used in FE simulations are listed in Supplementary Table 10.

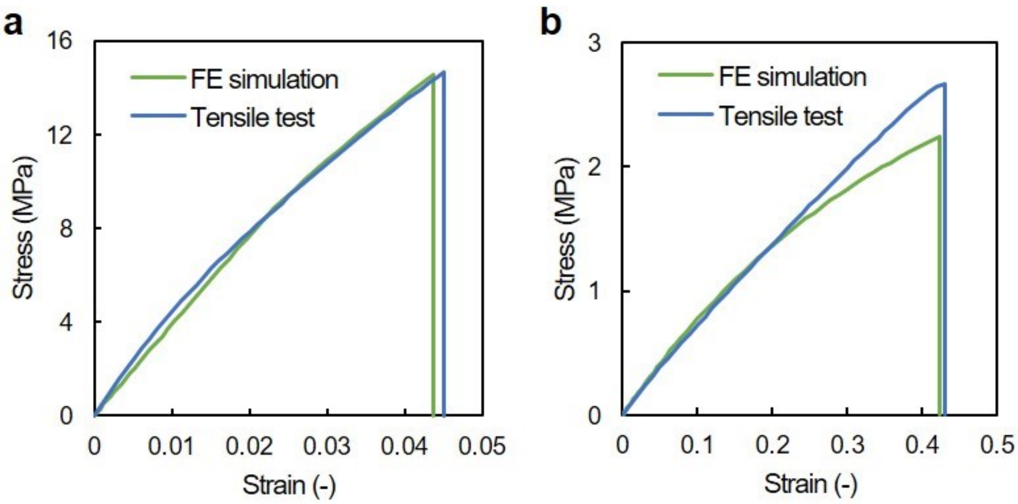

Supplementary Fig. 31 | Properties of the base material used in this work. a, TMPTA. **b**, Formlabs Flexible.

Supplementary Table 10 | Material properties of the base materials used in this study.

Material properties	TMPTA	Formlabs flexible
Density ρ_s (kg m^{-3})	1100	1100
Young's modulus E_s (MPa)	392.6	8.925
Yield strength σ_{ys} (MPa)	9.03	3.207
Strength at break σ_{fs} (MPa)	14.45	3.207
Strain at break ε_{fs} (-)	0.044	0.424
Poisson's ratio v_s (-)	0.3	0.48

Supplementary Note 12. Applicability of our ML pipeline to other mechanical loadings

In the manuscript, we demonstrated the inverse design of metamaterials for various target uniaxial compressive responses as a representative application of the ML pipeline. The ML pipeline adopts the stress-strain curve as the input, which can also describe mechanical behaviors under other types of loading such as tension, shear, torsion, and bending or external stimuli (e.g., loading direction, loading rate, temperature). We envision that the presented ML pipeline can be readily extended to inverse-design other mechanical behaviors, since the stress-strain curves used herein as input could represent other types of loading such as tension, shear, torsion, and bending, or different strain rate conditions.

In detail, the ML pipeline can be applied to inverse-design of metamaterials that replicates other mechanical responses individually (Supplementary Fig. 32). This can be achieved by adopting a collection of training datasets (i.e., training databank), wherein each of which contains a specific loading case (e.g., tensile, compressive, bending, shear) linked to their corresponding metamaterial topology. The sample size for each dataset is expected to be similar to the one used in the manuscript. A user can then pass a desired response (in form of stress-strain curve) along with a newly introduced input variable (Π), classifying a loading type, onto the ML pipeline. For instance, when Π is 1, a tensile dataset is selected from the databank to train the ML pipeline. Although, during training, the hyperparameters of the neural network models need to be tuned to specifically describe the nature/complexity of the mapping relationship of interest (i.e., curve features-to-metamaterial topology) so as to maximize the validation accuracy, we expect the structure of the

present ML pipeline and the training process to remain the same as described in the present manuscript. Once training is done, the target stress-strain curve of interest can then be inverse-designed following the same process as that introduced herein for the compressive loading case.

Beyond individual loading cases, the ML pipeline can be further modified to inverse-design metamaterials that satisfy multiple mechanical behavior inputs simultaneously (i.e., “multi-objective” inverse design). Supplementary Fig. 33 illustrates an example of a modified ML pipeline that can inverse-design some metamaterials that simultaneously feature targeted tensile and shear stress-strain curves. The modified ML pipeline is structured so that the inverse design module now involves with two surrogate forward validation modules where each of the forward modules aims at predicting one of these two stress-strain curves associated with both of the loading cases. For training, datasets from the databank corresponding to loading types of the target curves are first selected by utilizing the loading type identifier input (Π) described by an array (e.g., [1,4]). These datasets are then used to create a joint training set, which maps a metamaterial topology to multiple responses (e.g., tensile and shear responses). During training, the hyperparameters will need to be adjusted to achieve satisfactory validation accuracy. Once the target curves are fed into the modified ML pipeline, the inverse design module predicts five sets of design candidates, which will be passed to the forward validation module to estimate the response of the design candidates, as described in the manuscript. For each design candidate, the total normalized root-mean-square error (NRMSE) value (calculated as the weighted sum of the NRMSE associated with each loading case) can be used to select the optimal design. Indeed, a detailed future study is required to realize how to (1) combine the joint dataset, (2) determine the training data size required for each loading

type, and (3) identify the weight assigned to each loading case to ensure that none of the mechanical behaviors of interest dominates the others in the total cost function during the optimal design selection. Furthermore, similar to the design rules used for the plottable compressive stress-strain curve input described in the manuscript (as well as Supplementary Note 3), additional design rules may be necessary to simultaneously inverse-design other mechanical responses.

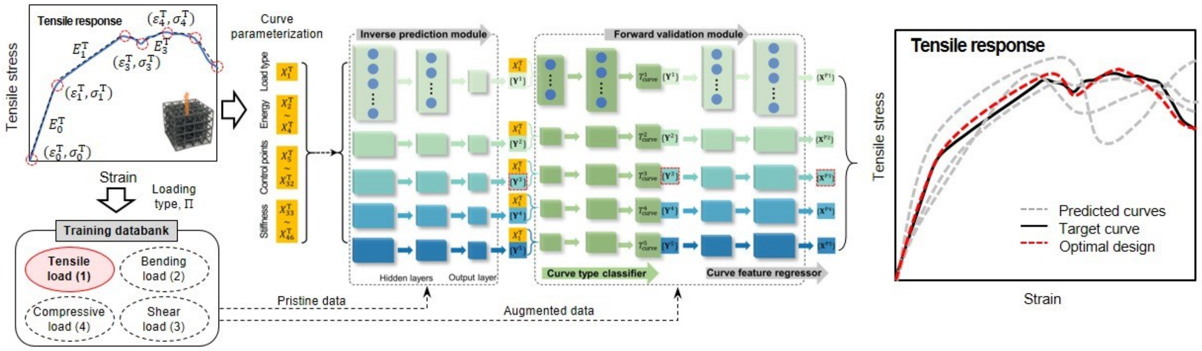

Supplementary Fig. 32. Illustration of the ML pipeline for the inverse design of other single mechanical responses. The input consists of a target curve and a new input variable classifying a loading type, denoted by *Π*. This variable guides the ML pipeline to select a relevant dataset from the training databank. The inverse prediction and forward validation modules are then trained by the selected data, wherein the hyperparameters of all the neural networks need to be tuned for each specific mechanical response. Once both modules are trained, the rest of the prediction process for the target curve remains the same as described in the manuscript.

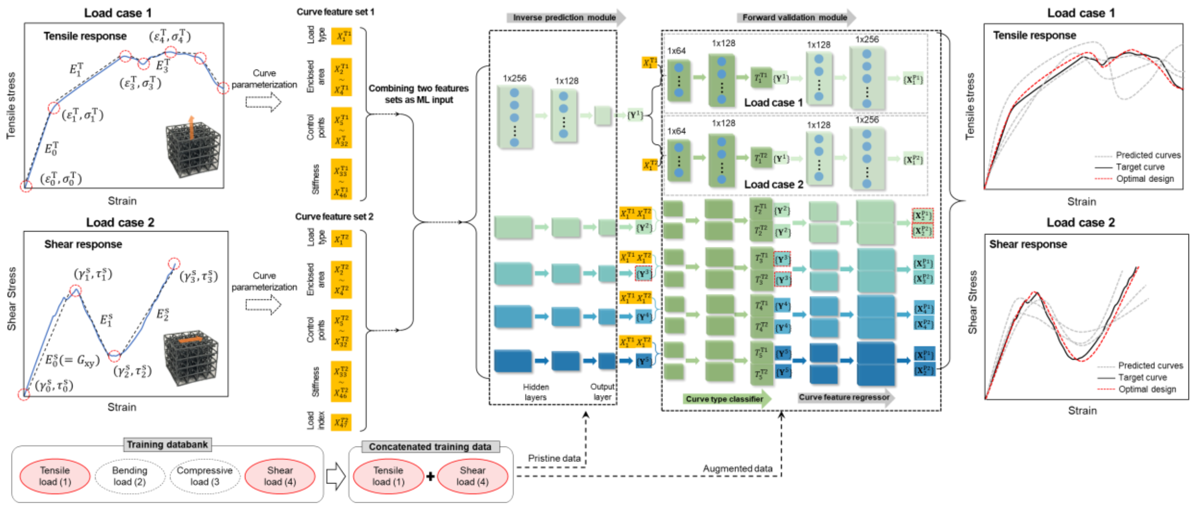

Supplementary Fig. 33. Example of the modified ML pipeline for the simultaneous inverse design of multiple mechanical responses. The target stress-strain curves associated with the loading conditions of interest are parameterized into two curve feature sets $\{\mathbf{X}^{\mathrm{T}i}\}$ ($i = 1, 4$, denoting the loading type identifier), which is then combined and used as input for the ML framework. The inverse prediction module predicts five sets of design candidates $\{\mathbf{Y}^k\}$ (described by the cell type (T_{cell}), characteristic angle (ϕ), and radius-to-length ratio (r_1/L_1)), where k ranges from 1 to 5. These design candidates are then passed to the forward validation module, where each inverse neural network model is linked with two surrogate models (each contains a curve type classifier and a curve feature regressor), to estimate the response $\{\mathbf{X}_k{}^{\mathrm{P}i}\}$ of the design candidates. For each design candidate, the total NRMSE value (calculated as the weighted sum of the NRMSE associated with each loading case) is used to select the optimal design. The weight assigned to each loading case would need to be adjusted to ensure that none of the mechanical behaviors of interest dominates the others in the total cost function.

Supplementary References

1 Gibson, I. & Ashby, M. F. The mechanics of three-dimensional cellular materials. *Proceedings of the royal society of London. A. Mathematical and physical sciences* **382**, 43-59 (1982).

2 Hashin, Z. & Shtrikman, S. A variational approach to the theory of the elastic behaviour of multiphase materials. *Journal of the Mechanics and Physics of Solids* **11**, 127-140 (1963).

3 Suquet, P. Overall potentials and extremal surfaces of power law or ideally plastic composites. *Journal of the Mechanics and Physics of Solids* **41**, 981-1002 (1993).

4 Oliphant, T. *A guide to NumPy*. (Trelgol Publishing USA, 2006).

5 Virtanen, P. *et al.* SciPy 1.0: fundamental algorithms for scientific computing in Python. *Nat. Methods* **17**, 261-272 (2020).

6 McKinney, W. pandas: a foundational Python library for data analysis and statistics. *Python for High Performance and Scientific Computing* **9**, 14 (2011).

7 Schutt, K. *et al.* How to represent crystal structures for machine learning: Towards fast prediction of electronic properties. *Phys. Rev. B* **89**, 205118 (2014).

8 Savitzky, A. & Golay, M. Smoothing and differentiation of data by simplified least squares procedures. *Anal. Chem.* **36**, 1627-1639 (1964).

9 Tancogne-Dejean, T., Diamantopoulou, M., Gorji, M. B., Bonatti, C. & Mohr, D. 3D Plate-Lattices: An Emerging Class of Low-Density Metamaterial Exhibiting Optimal Isotropic Stiffness. *Advanced Materials* **30**, 1803334 (2018).

10 Crook, C. *et al.* Plate-nanolattices at the theoretical limit of stiffness and strength. *Nature communications* **11**, 1-11 (2020).

11 Onck, P., Andrews, E. & Gibson, L. Size effects in ductile cellular solids. Part I: modeling. *International Journal of Mechanical Sciences* **43**, 681-699 (2001).

12 Andrews, E., Gioux, G., Onck, P. & Gibson, L. Size effects in ductile cellular solids. Part II: experimental results. *International Journal of Mechanical Sciences* **43**, 701-713 (2001).

13 Morrish, S., Pedersen, M., Wong, K., Todd, I. & Goodall, R. Size effects in compression in Electron Beam Melted Ti6Al4V diamond structure lattices. *Materials Letters* **190**, 138-142 (2017).

14 Chawla, N. V., Bowyer, K. W., Hall, L. O. & Kegelmeyer, W. P. SMOTE: Synthetic Minority Over-sampling Technique. *Journal of Artificial Intelligence Research* **16**, 321-357 (2002). https://doi.org:10.1613/jair.953

15 Mazur, M. *et al.* Deformation and failure behaviour of Ti-6Al-4V lattice structures manufactured by selective laser melting (SLM). *The International Journal of Advanced Manufacturing Technology* **84**, 1391-1411 (2016).

16 Liu, L., Kamm, P., García-Moreno, F., Banhart, J. & Pasini, D. Elastic and failure response of imperfect three-dimensional metallic lattices: the role of geometric defects induced by Selective Laser Melting. *Journal of the Mechanics and Physics of Solids* **107**, 160-184 (2017).

17 Hitzler, L. *et al.* On the anisotropic mechanical properties of selective laser-melted stainless steel. *Materials* **10**, 1136 (2017).

18 Xiao, Z. *et al.* Evaluation of topology-optimized lattice structures manufactured via selective laser melting. *Materials & Design* **143**, 27-37 (2018).

19 Mohri, M., Rostamizadeh, A. & Talwalkar, A. *Foundations of machine learning*. (MIT press, 2018).

20 Pilania, G., Gubernatis, J. E. & Lookman, T. Multi-fidelity machine learning models for accurate bandgap predictions of solids. *Computational Materials Science* **129**, 156-163 (2017).

21 Patra, A. *et al.* A multi-fidelity information-fusion approach to machine learn and predict polymer bandgap. *Computational Materials Science* **172**, 109286 (2020).

22 Alasadi, S. A. & Bhaya, W. S. Review of data preprocessing techniques in data mining. *Journal of Engineering and Applied Sciences* **12**, 4102-4107 (2017).

23 Henning, E. & Milani, T. In-shoe pressure distribution for running in various types of footwear. *J. Appl. Biomech.* **11**, 299-310 (1995).

24 Sutrisno, A. & Braun, D. J. How to run 50% faster without external energy. *Sci. Adv.* **6**, eaay1950 (2020).

25 Schaedler, T. *et al.* Designing Metallic Microlattices for Energy Absorber Applications. *Adv. Eng. Mater.* **16**, 276-283 (2014).

26 Lai, C. & Daraio, C. Highly porous microlattices as ultrathin and efficient impact absorbers. *Int. J. Impact Eng.* **120**, 138-149 (2018).

27 Frenzel, T., Findeisen, C., Kadic, M., Gumbsch, P. & Wegener, M. Tailored buckling microlattices as reusable light-weight shock absorbers. *Adv. Mater.* **28**, 5865-5870 (2016).

28 Correa, D. *et al.* Negative stiffness honeycombs for recoverable shock isolation. *Rapid Prototyp. J.* (2015).

29 Maskery, I. *et al.* An investigation into reinforced and functionally graded lattice structures. *J. Cell. Plast.* **53**, 151-165 (2017).

30 Chen, S. *et al.* Elastic carbon foam via direct carbonization of polymer foam for flexible electrodes and organic chemical absorption. *Energy Environ. Sci.* **6**, 2435-2439 (2013).

31 Moss, W. & King, M. Impact response of US Army and National Football League helmet pad systems. (Lawrence Livermore National Lab CA, 2011).
