# Supplementary material for: Rapid inverse design of metamaterials based on prescribed mechanical behavior through machine learning
Source: Nat Commun. 2023 Sep 18;14:5765. doi: 10.1038/s41467-023-40854-1 (PMC10505607; doi:10.1038/s41467-023-40854-1)
Supplement: Supplementary file 3 — Description of Additional Supplementary Files [file 41467_2023_40854_MOESM3_ESM.pdf]

Inventory of Supporting Information

Filename: Supplementary Movie 1
Description: Architectural evolution of the cubic symmetric, strut-based unit cells.

Filename: Supplementary Movie 2
Description: In situ movie of the inversely designed sample shown in Fig. 5d in response to uniaxial cyclic compression.

Filename: Supplementary Movie 3
Description: In situ movie of the inversely designed sample shown in Fig. 5e in response to uniaxial cyclic compression.

Filename: Supplementary Movie 4
Description: In situ movie of the inversely designed sample shown in Fig. 5f in response to uniaxial cyclic compression.

Filename: Supplementary Information
Description: The supplementary information of the main manuscript.
